# Emerging Biomimetic Drug Delivery Nanoparticles Inspired by Extracellular Vesicles

**DOI:** 10.1002/wnan.70025

**Published:** 2025-08-21

**Authors:** Viswanathan Sundaram, Santosh Aryal

**Affiliations:** ^1^ Department of Pharmaceutical Sciences and Health Outcomes, The Ben and Maytee Fisch College of Pharmacy The University of Texas at Tyler Tyler Texas USA

**Keywords:** drug delivery, exosomes, extracellular vesicles, hybrid vesicles, liposomes

## Abstract

Nanoparticles (NPs) made up of cellular components such as extracellular vesicles (EVs) with a biomimetic outlook have emerged as a revolutionary approach in nanomedicine, providing significant benefits for targeted drug administration, immunotherapy, monitoring therapeutic response, and diagnostic applications. Utilizing the distinctive characteristics of natural cell membranes, membrane proteins, and cellular contents, these biomimetic NPs acquire essential biological functions from their source and biogenesis, including immune evasion, extended circulation, and target recognition, rendering them optimal candidates for therapeutic applications. This review offers a comprehensive examination of the methodologies of EVs infused with synthetic NP systems with the goal of overcoming their respective shortcomings. For instance, EVs are biogenic with cellular targeting features, but their isolation yield is limited, and their structural and colloidal stability are weak. Whereas, we have decades of experience in the mass production of highly stable synthetic NPs, they lack cellular targeting features. Therefore, the integration of these two systems as a single entity in the field of nanomedicine has gained significant attention. In this review, we emphasized the variety of EVs sources, such as erythrocytes, leukocytes, cancer cells, and stem cells, each providing unique biological benefits. Critical procedures encompassing EV's separation, coating processes, and material integration were examined while addressing the issues, including scalability, membrane stability, and preservation of functionality. Additionally, their promise in customized medicine is analyzed, highlighting their immediate medical applications. This review seeks to elucidate the existing methodologies, their constraints, and prospective advancements in the creation of EV‐derived biomimetic NPs for clinical use.

This article is categorized under:

Nanotechnology Approaches to Biology > Nanoscale Systems in Biology
Therapeutic Approaches and Drug Discovery > Nanomedicine for Oncologic Disease

## Graphical abstract

An overview of various nanoparticles reviewed in this article and their mechanism of cellular targeting.

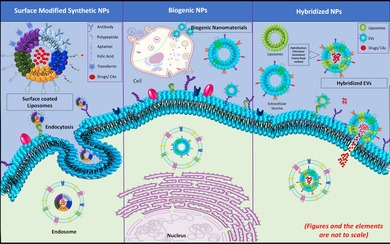

**Abbreviations**
AD‐MSCs: adipose tissue‐derived MSCs
ADP: adenosine diphosphate
AF‐MSCs: amniotic fluid‐derived MSCs
ATRA: all‐*trans*‐retinoic acid
BCL‐2: B‐cell lymphoma 2
BMECs: brain microvascular endothelial cells
BM‐MSCs: bone marrow‐derived MSCs
CAFs: cancer associated fibroblasts
Cas12a: CRISPR‐associated protein 12a
Cas13a: CRISPR‐associated protein 13a
Cas9: CRISPR‐associated protein 9
CCR5: cysteine–cysteine chemokine receptor 5
CD47: cluster of differentiation 47
CFSE: carboxy fluorescein succinimidyl ester
CK‐MB: creatine kinase‐MB
CLD: clodronate
CpG: cytosine‐phosphate‐guanine
CRISPR: clustered regularly interspaced short palindromic repeats
CT: computed tomography
CTLA‐4: cytotoxic T‐lymphocyte‐associated antigen 4
CTX: cetuximab
DCs: dendritic cells
DDS: drug delivery systems
DiI: 1,1′‐dioctadecyl‐3,3,3′,3′‐tetramethylindocarbocyanine
DLS: dynamic light scattering
DNA: deoxyribonucleic acid
DOX: doxorubicin
DOXIL: liposomal doxorubicin
DP‐MSCs: dental pulp‐derived MSCs
EGFR: epidermal growth factor receptor
EGFRvIII: epidermal growth factor receptor variant III
ELISA: enzyme‐linked immunosorbent assay
eMSCs: endometrium‐derived MSCs
EpCAM: epithelial cell adhesion molecule
EPM: EVs loaded with paclitaxel albumin and melanin
EPR: enhanced permeability and retention
EVs: extracellular vesicles
FR+: folate receptor‐positive
GdL: gadolinium lipid
GdMEVs: glioblastoma‐derived small extracellular vesicles
GFP: green fluorescent protein
GPx: glutathione peroxidase
HAEC: human aortic endothelial cells
HCAEC: human coronary artery endothelial cells
hCMEC/D3: human cerebral microvascular endothelial cell line
HDLEC: human dermal lymphatic endothelial cells
HEK293: human embryonic kidney 293 cells
HER2: human epidermal growth factor receptor 2
HEs: hybrid exosomes
HFFs: human foreskin fibroblasts
HIV: human immunodeficiency virus
HMEC‐1: human mammary epithelial cells
HPAEC: human pulmonary artery endothelial cells
HPLC: high‐performance liquid chromatography
HSP70: heat shock protein 70
HSP90: heat shock protein 90
HSPGs: heparan sulfate proteoglycans
HSV: herpes simplex virus
HUC‐MSC: human umbilical cord mesenchymal stem cells
HUVECs: human umbilical vein endothelial cells
ICP‐MS: inductively coupled plasma mass spectrometry
IDO: indoleamine 2,3‐dioxygenase
IL‐10: interleukin‐10
IL‐1Ra: interleukin 1 receptor antagonist
IL‐4Ra: interleukin‐4 receptor subunit alpha
iPSC‐MSCs: induced pluripotent stem cell‐derived MSCs
KRAS: Kirsten rat sarcoma viral oncogene homolog
LEVs: liposome hybridized EVs
MEFs: mouse embryonic fibroblasts
MenMSCs: menstrual blood‐derived MSCs
MFHE: membrane fusion‐based hybrid exosomes
miR: miRNAs
miRNAs: microRNA
MRI: magnetic resonance imaging
MSCs: mesenchymal stem cells
MVECs: microvascular endothelial cells
NHDFs: normal human dermal fibroblasts
NIR: near‐infrared
NK: natural killer cells
NPs: nanoparticles
NTA: nanoparticle tracking analysis
PA: paclitaxel albumin
PAI: plasminogen activator inhibitor
PCSK9: proprotein convertase subtilisin/kexin type 9
PDAC: pancreatic ductal adenocarcinoma
PDI: polydispersity index
PEG: polyethylene glycol
PET: positron emission tomography
P‐MSCs: placenta‐derived MSCs
PRP: platelet‐rich plasma
PSA: prostate‐specific antigen
PSMA: prostate‐specific membrane antigen
PTX: paclitaxel
PTX‐HEs: PTX‐loaded hybridized exosomes
r1: longitudinal relaxivity 1
RES: reticuloendothelial system
RGD: arginine–glycine–aspartate
RNA: ribonucleic acid
ROS: reactive oxygen species
SELEX: systematic evolution of ligands by exponential enrichment
sEVs: small EVs
sgRNA: single guide RNA
siRNA: small interfering RNA
SM‐MSCs: synovial membrane MSCs
SOD: superoxide dismutase
SPECT: single photon emission computed tomography
SpHL‐DOX‐Fol: folate‐coated doxorubicin‐loaded pH‐sensitive liposome
SPIONs: superparamagnetic iron oxide nanoparticles
SR‐B1: Scavenger receptor class B‐ type I
TB: tuberculosis
TEM: transmission electron microscopy
TEVs: tumor‐derived EVs
Tf: transferrin
TfR: transferrin receptors
TGF‐β: transforming growth factor‐beta
TNF‐α: tumor necrosis factor‐alpha
TPGS: d‐α‐tocopheryl polyethylene glycol 1000 succinate
TRegs: regulatory T cells
UC‐MSCs: umbilical cord‐derived MSCs
US FDA: Food and Drug Administration, United States

## 1 Introduction

Nanobiotechnology enables molecular‐level material manipulation for biomedical applications with the goal of creating nontoxic bioactive nanodevices that are selective to the intended biological location. The advantage of utilizing nanomaterials as drug delivery systems (DDS) resides in their nano size, allowing them to pass through biological barriers and constricted capillaries, ultimately reaching specific organs, tumors, or individual cells (Sun et al. 2014). The use of nanotechnological materials for the prevention, monitoring, and intervention of diseases is termed nanomedicine. Nanomedicine is designed to incorporate imaging, therapy, diagnosis, repair, and regeneration modalities for medical applications. Over the past decades, nanomedicine has been developing into a vital branch in the medical field (Anjum et al. 2021; Morrow et al. 2007). The commonly available conventional classes of NPs in the nanomedicine field are liposomes, biodegradable polymers, hydrogel nanocomposites, semiconductor nanomaterials, magnetic nanomaterials, solid lipid NPs, metal NPs, polymer nanocomposites, dendrimers, inorganic NPs, and micelles (Ferrel et al. 2021; Hajfathalian et al. 2024; Huang, Zhou, et al. 2022; Jackson et al. 2017; Jiang et al. 2021; Kass and Nguyen 2022; Lee and Thompson 2017; Luzuriaga et al. 2021; Movassaghian et al. 2015; Del Rahmani Bakhshayesh et al. 2023). Conventional NPs become the researcher's first choice because of their in vitro and in vivo drug stability, therapeutic efficacy, and ease of surface modification to install unique properties (Greish 2018). The NPs are emerging as the most advantageous tool in nanomedicine based on the following factors. The tiny size of the NPs allows efficient absorption and solubility of the conventional drugs, which often need a combination of various organic solvents and higher doses. NPs can be designed to minimize adverse side effects on the human body (Sharma and Alam 2023). After entering the blood circulation, the NPs can interact with key molecules, leading to the formation of bio‐corona and further interaction with organelles, which can lead to off‐target effects and even cell death (Ajith et al. 2022). Therefore, biomimetic modification of NPs would result in enhanced targeting, lower toxicity, and may maintain the desired pharmacokinetics (Jeevanandam et al. 2018; Pitchaimani et al. 2018, 2019; Tikhonov et al. 2024).

Biomimetic NPs engineered by the fusion of EVs and synthetic NPs are an emerging and innovative platform that mimics the biological characteristics and functions of native cells. These NPs provide enhanced biocompatibility, exceptional target specificity, prolonged retention time, and minimum unwanted immune reactions (Alimohammadvand et al. 2024; Hu et al. 2023; Sarkar Lotfabadi et al. 2024; Sherawata et al. 2023; Tikhonov et al. 2024). These NPs receive significant attention because of their superior biocompatibility and reduced unwanted immune responses in comparison to other NPs (Chakraborty et al. 2023). These NPs have the capability of inducing several desired biological effects due to their inherent richness in cell‐specific functionality (Khojini et al. 2023). They can be employed as drug delivery system carriers with high specificity and efficiency (Manika and Pandey 2023). Additionally, biomimetic NPs have been explored for their applications in various fields such as cancer immunotherapy, bioanalysis, and biomedical engineering. Overall, biomimetic NPs hold great promise for the development of advanced biomaterials with specialized biological functions. These NPs are designed to emulate the structural and functional characteristics of natural biomolecules and organisms, allowing for unique properties and applications. The field is leveraging the principles of biology to create innovative and versatile materials. They have been used in a wide range of applications, including drug delivery, gene therapy, tissue engineering, and sensing. One notable application of biomimetic NPs is in tumor‐specific drug delivery.

Extracellular vesicles (EVs) are one of the well‐known and emerging candidates in nanomedicine to derive biomimetic NPs (Du, Guan, et al. 2023; Mondal et al. 2024). EVs are nanosized, lipid bilayer‐bounded bodies that facilitate intracellular communication, impacting cell response. Almost all types of eukaryotic and prokaryotic cells release these EVs into the extracellular space through major molecular mechanisms such as the endosomal pathway (exosome formation), direct plasma membrane budding (microvesicle formation), and apoptotic pathway (release of apoptotic bodies) (Maas et al. 2017; Mathieu et al. 2019; van Niel et al. 2018; Xiang et al. 2024). The mechanistic action for intracellular communication of EVs facilitates transport systems for cell‐derived bioactive molecules, including proteins, lipids, RNA, DNA, and metabolic intermediate molecules, to the recipient cell from the donor cell. These EVs are commonly heterogeneous particles that are classified into different types based on their size and functions as exosomes (30–150 nm), microvesicles (0.1–2 μm), and apoptotic bodies (1–5 μm). Based on their cellular origin, these are classified; for example, those originating from cancer cells are called oncosomes (Liu 2024; Xiang et al. 2024). These EVs are extensively found in nearly all biological fluids such as blood, urine, synovial fluid, saliva, breast milk, and cerebrospinal fluid. The protein markers of EVs play a crucial role in intracellular communication and also help to assess and characterize the EVs. The commonly available protein markers in the EVs include tetraspanins (CD9, CD63, CD81), heat shock proteins (HSP70), Alix, and tumor susceptibility gene (TSG101) (Aloi et al. 2024; Das 2024; Papoutsoglou and Morillon 2024; Yu et al. 2024; Zhang, Wu, et al. 2022).

The EVs are entering different recipient cells by various endocytic mechanisms such as caveolin‐mediated uptake, clathrin‐dependent endocytosis, phagocytosis, macro‐pinocytosis, and lipid raft‐mediated internalization (Aloi et al. 2024; Geng et al. 2024; Hirosawa et al. 2025; Xiang et al. 2024). The surface proteins and glycoproteins of EVs and cells are the primary factors that direct the uptake mechanisms based on their activities (Du et al. 2024; Ginini et al. 2022; Mulcahy et al. 2014; Williams et al. 2019). The functional proteins present on the surface of the EVs facilitate therapeutic and diagnostic applications, which are guided by parental cell properties that they acquired. For example, EVs generated from immune cells such as macrophages, T‐cells, and natural killer cells were found to target inflammatory cells. Similarly, EVs derived from tumors enhance communication between cancer cells and other cells within the tumor microenvironment, such as endothelial cells, fibroblasts, and immune cells, thus influencing cancer growth and immunological responses (Bao et al. 2022; Tai et al. 2019; Yu et al. 2024). They can alter the tumor microenvironment by facilitating angiogenesis, immune evasion, and the development of premetastatic niches, promoting cancer progression (Biray Avci et al. 2024; Li, Zheng, et al. 2024; Mir et al. 2024; Vasconcelos et al. 2019). The tumor cells derived EVs transport diverse macromolecules, such as DNA, RNA, proteins, and lipids, capable of imparting aggressive phenotypic traits and drug‐resistant properties to other cancer cells (Hu et al. 2022; Mir et al. 2024; Willms et al. 2018; Wortzel et al. 2024; Xiong et al. 2024; Zuo et al. 2024). Owing to their distinctive molecular signatures, tumor‐derived EVs serve as significant diagnostic and predictive indicators in liquid biopsies, facilitating real‐time surveillance of cancer development and treatment response (Dabral et al. 2024; Liao et al. 2024; Rahbarghazi et al. 2019; Tai et al. 2019). EVs have demonstrated the capability to identify early‐stage neoplastic tissues and circulating tumor cells, which may be employed for early identification and targeted administration of treatment medicines to inhibit tumor progression (Garofalo et al. 2021). Their capacity to transport and administer oncogenic chemicals renders them significant instruments in precision medicine, with current investigations focusing on their potential for early detection and targeted therapy (Pagotto et al. 2023; Rahbarghazi et al. 2019; Tai et al. 2019). These unique properties of tumor cell‐derived EVs can be advantageous in precision drug delivery when appropriately re‐engineered with chemotherapeutics and other synthetic NPs.

Liposomes are synthetic active tools for efficient drug delivery, which have already advanced to the clinics (Eugster et al. 2024; Hamad et al. 2024; Liu et al. 2022; Nsairat et al. 2022; Yi et al. 2022). Liposomes are spherical‐shaped vesicles made of cholesterol and phospholipids with the ability to encapsulate both hydrophilic and hydrophobic materials in the core and bilayer surface of the lipids (Andra et al. 2022; Lombardo and Kiselev 2022). Since their discovery in the 1960s, liposomes have been extensively studied and utilized in various medical applications, including cancer therapy, vaccine delivery, and treatment of infectious diseases (Bozzuto and Molinari 2015; Hsu et al. 2023; Karunakaran et al. 2023; Mehta, Bui, et al. 2023; Rommasi and Esfandiari 2021). Understanding the fundamental concepts of liposomes, which include their formation, structure, and release kinetics, is crucial for designing effective experiments and products utilizing these lipid‐based carriers. The design of liposomes involves careful consideration of their size, composition, surface charge, and bilayer fluidity to optimize drug delivery. Liposomes can control drug release, prevent drug degradation, and alter drug pharmacokinetics, which is beneficial for treating diseases like cancer and infections. However, they lack disease‐specific targeting capabilities. Addressing these disadvantages is crucial for expanding their applications in various fields.

The delivery systems of EVs and liposomes have numerous advantageous functions as an envelope for drugs; on the other hand, their shortcomings lack their efficient drug delivery effect. These include that EVs are expert in cellular targeting but are limited in their isolation yield, and their structural and colloidal stability is weak, whereas liposomes lack targeting features but are well established as NP drug delivery systems. The uniqueness of these two systems is their similarities in vesicular structure with an aqueous core. Therefore, the hybridization of EVs and liposomes is an excellent approach to overcome their respective shortcomings. These hybrid systems aim to leverage the natural properties of EVs and the customizable features of synthetic liposomes to enhance therapeutic delivery. EV‐based hybridized biomimetic NPs offer a novel strategy by merging the intrinsic characteristics of artificial nanocarriers with the capabilities of biological cell membranes. The major classes of biomimetic NPs were synthesized based on cellular membranes and EVs because these NPs easily mimic the target cells and resemble the characteristics of cell membranes (Ferrel et al. 2021; Pitchaimani et al. 2018; Qiu et al. 2024; Sushnitha et al. 2020). The principle behind hybridization is that through this integration, the parent cell membrane proteins and lipids are infused into the NPs to feature biocompatibility, targeted drug delivery, and stealth properties. Among the synthetic NPs, liposomes are one of the notable NPs that are easily mimicked by cell membranes. EVs are naturally biocompatible and possess inherent targeting capabilities due to their protein‐rich lipid bilayer. When combined with liposomes, these hybrid systems exhibit enhanced cellular uptake, immuno‐evasive properties, and the ability to cross biological barriers, which are significant improvements over purely synthetic systems (Rayamajhi et al. 2019; Rodríguez and Vader 2022; Sulthana et al. 2024). Hybrid NPs, such as EV‐liposome hybrids, have shown improved delivery of therapeutic agents, diagnostic agents, and siRNA to target cells (Evers et al. 2022; Kim, Park, et al. 2024; Rayamajhi et al. 2019; Sulthana et al. 2024). These hybrids encapsulate siRNA effectively and demonstrate altered cellular uptake and gene‐silencing efficacy compared to traditional liposomes, making them a potent delivery system (Du, Guan, et al. 2023; Evers et al. 2022; Walker et al. 2019).

**FIGURE 1:**
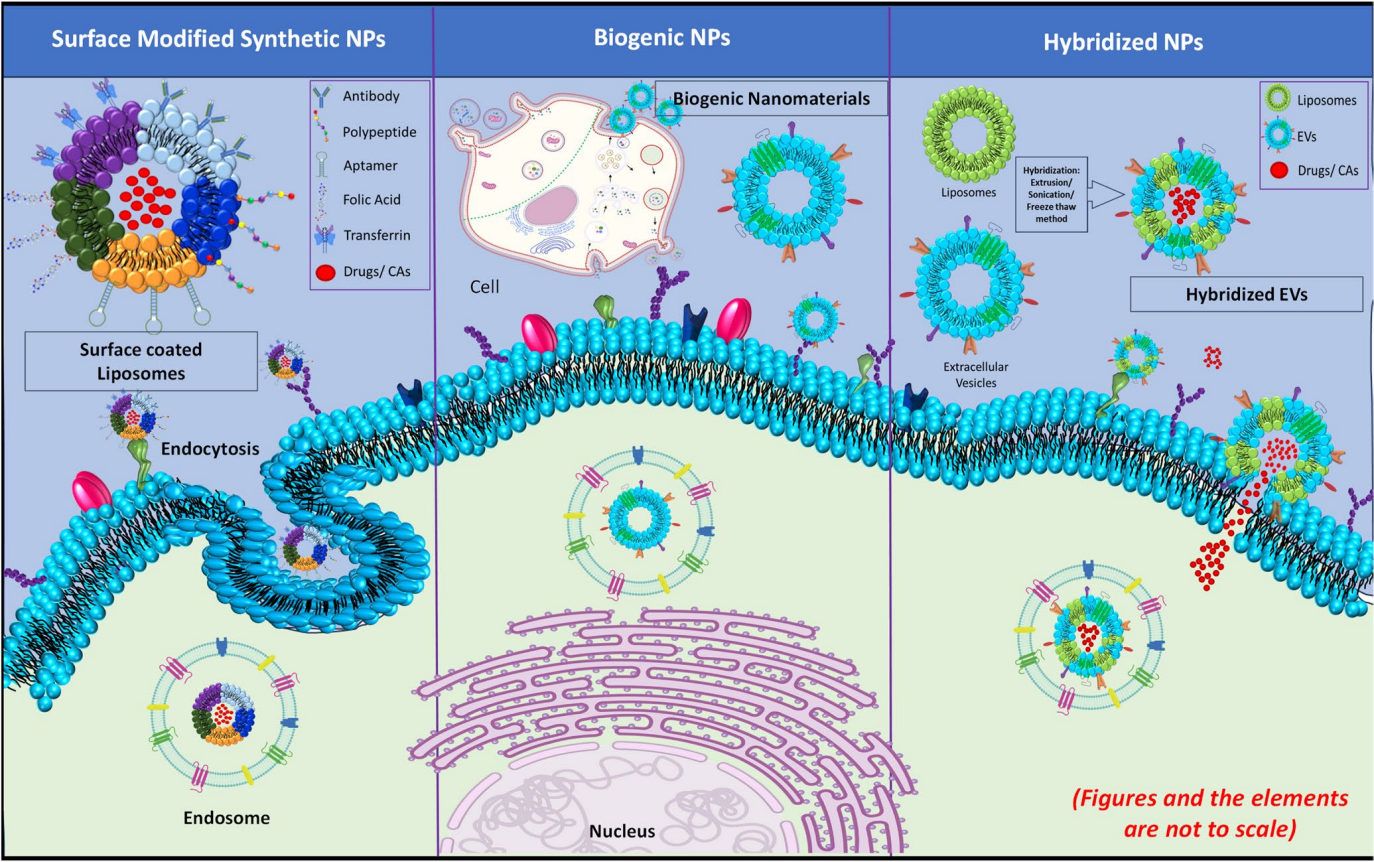
An overview of strategic nanoparticles reviewed in this article and their interaction with cancer cells. This schematic explains the structure of various NPs discussed and their mechanism of cellular targeting.

This overall review focused on addressing the isolation and synthetic approaches of membrane‐based biomimetic NPs with a focus on EVs and liposome‐integrated systems (Figure 1). We are highlighting their ability to interact with their biological targets.

## 2 Extracellular Vesicles (EVs)

**FIGURE 2:**
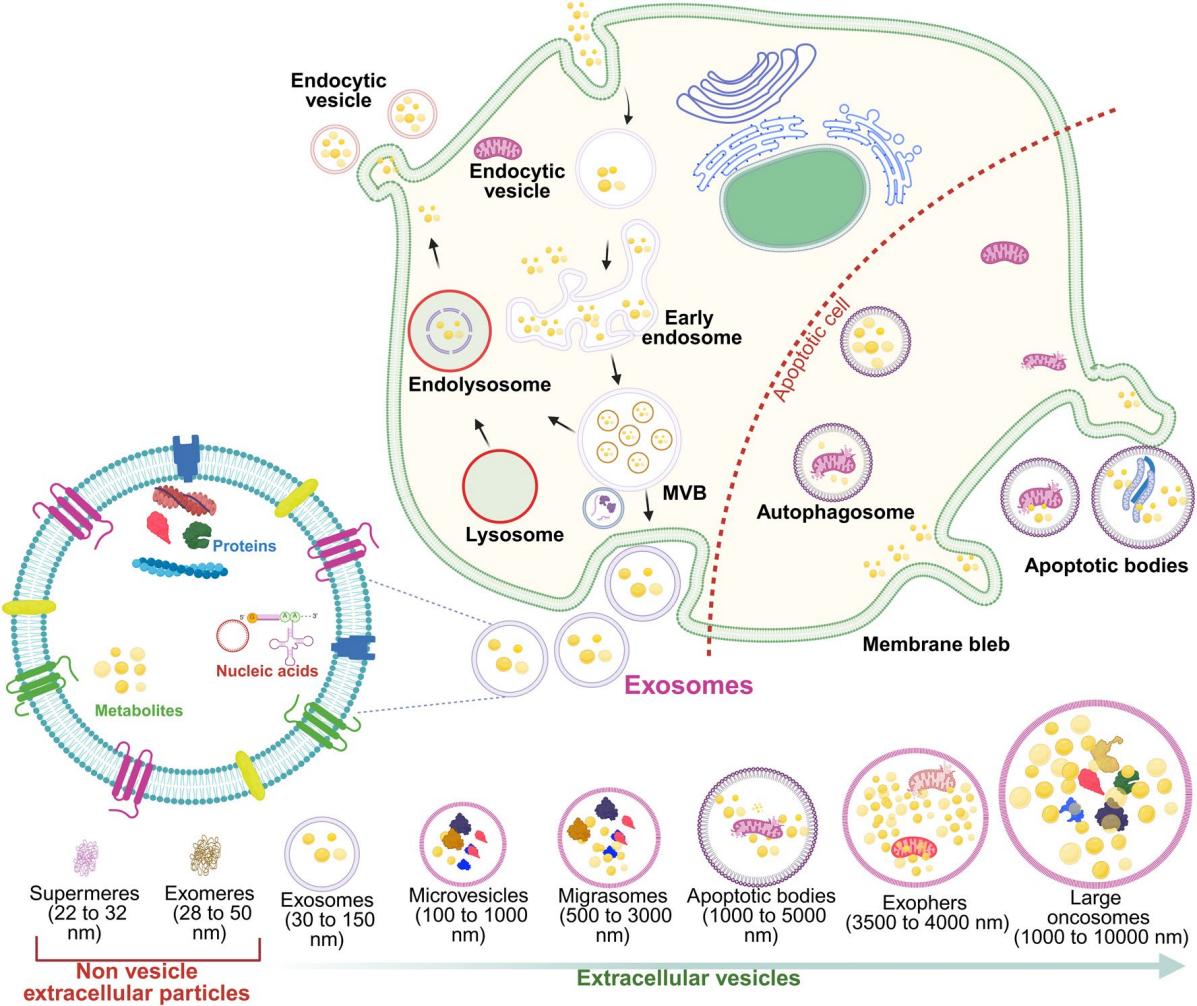
Biogenesis of different types of EVs. EVs are biogenically synthesized by cells, and these are membrane‐bound architectures that help to transfer the biofunctional molecules such as proteins, lipids, and nucleic acids. They actively participated in various pathophysiological processes. The major types of EVs are exosomes, ectosomes, and apoptotic bodies.

EVs are phospholipid bilayer‐enclosed vesicles released by all cell types. They can be identified in tissue culture supernatants, blood, saliva, breast milk, cerebrospinal fluids, and malignant ascites. EVs are divided into three types based on their biogenesis: exosomes, microvesicles, and apoptotic bodies (Kalra et al. 2016; van Niel et al. 2018). EVs are stable in biofluids and organisms, and they can distribute over short and long distances, even penetrating the biological barrier. EVs are unique in protecting and delivering their internal cargo to target cells through ligand–receptor interactions. Previous research indicates that proteins on EVs' surfaces enhance cargo circulation and prolong circulatory half‐lives by enhancing membrane fusion with the targeted cells and inhibiting CD47‐mediated phagocytic clearance, hence improving the pharmacological features of EVs (Kamerkar et al. 2017). Cellular uptake of EVs depends on surface ligands like heparan sulfate proteoglycans (HSPGs) or recipient cell surface receptors like scavenger receptor class B, type 1 (SR‐B1) (Du et al. 2024). Recent research works appear to indicate that EVs are predisposed to certain organs, allowing for the targeted loading of cargo into the EVs to deliver into the recipient cells. Due to their nanoscale size, EVs can be efficiently transported through bodily fluids and biological barriers. Considering that this particular targeting ability can be meticulously regulated with higher efficiency, EVs will serve as an effective system for delivering therapeutic agents (Figure 2).

The clinical translation of extracellular vesicles (EVs) has experienced significant progress recently, with an increasing number of preclinical studies showcasing their promising ability as targeted therapeutic carriers, biomarkers, and immunological modulators. Significant advantages facilitating clinical translation include the ability of extracellular vesicles (EVs) to cross biological barriers, their reduced immunogenicity relative to synthetic nanoparticles, and the potential for the creation of surface ligands to improve tissue‐specific delivery (Ghodasara et al. 2023). Despite such advantages, the therapeutic utilization of EVs encounters considerable obstacles, including scalable production, variability in isolation techniques, reproducibility between batches, and regulatory categorization. Confronting these problems has emerged as a principal objective for biotech startups and academic spin‐offs globally, especially notable progress seen in the Far East countries. The major countries, including Japan, South Korea, Singapore, and China, have emerged as centers for advanced research and development of extracellular vesicles. Korean biotech businesses, including Eutilex and Curocell, are advancing extracellular vesicle‐based immunotherapies, whereas Japanese firms like Evox Therapeutics are concentrating on modified extracellular vesicles for targeted drug delivery (Claridge et al. 2021; Uddin et al. 2024; Wiklander et al. 2019). Singaporean firms such as Paragraf Therapeutics are investigating diagnostics based on extracellular vesicles, while Chinese companies like Everest Medicines are investing in scalable production technology for extracellular vesicles. These initiatives highlight the worldwide competition for the commercialization of EVs, focusing on GMP‐compliant manufacturing, precise cargo loading, and regulated biodistribution. The aggregate advancements of these biotech innovators feature the translational readiness of EVs and indicate their potential incorporation into next‐generation therapeutics and diagnostics (Stawarska et al. 2024).

EVs have a role in various physiological and pathological processes and have diverse biological activities. They play a role in complex biological processes like tumorigenesis, preparation of metastatic niches, elimination of cytotoxic drugs like cisplatin, inflammation, immune response modulation, angiogenesis, tissue repair, apoptosis, and homeostasis by transferring a wide range of molecules between cells (Adamczyk et al. 2023; Aguiar Koga et al. 2023; Ateeq et al. 2024; Chakraborty et al. 2023; Das 2024; Lin et al. 2024; Lin et al. 2024; Liu et al. 2024; Oliva 2023). Since their composition reflects parental cell status at production, they are attractive diagnostics. EVs are persistent in many bodily fluids, making them potential biomarker reservoirs. Liquid biopsies with circulating EVs could evaluate patient prognosis, disease progression, and medication response. EVs also paracrinally regulate cell phenotypes, differentiation, and recruitment (Bernáth‐Nagy et al. 2024; Guo et al. 2024; Malaguarnera and Cabrera‐Pastor 2024; Zhao and Huang 2024). Unlike stem cell therapies, stem cell‐derived EVs overcome some limitations, such as immune rejection and tumorigenic potential, which may make them a better therapeutic tool than stem cell therapy (Hur et al. 2020). Although EVs cannot self‐replicate, they may be safer than stem cell transplantation in regenerative medicine (de Jong et al. 2014; Li, Ji, et al. 2024; Romano et al. 2020; Tryfonidou 2024). EVs carry a wide range of biological compounds across biofluids with cellular selectivity, making them promising medication delivery vehicles. Recent proposals include putting imaging tracers (for diagnostics) and therapeutic chemicals into EVs to create an EV‐based theranostic delivery platform (Pitchaimani et al. 2016; Rakshit and Pal 2024; Wu et al. 2021; Zhang et al. 2023). Many preclinical and clinical research studies are validating these prospective applications (Ciferri et al. 2021; Kumar et al. 2024; Mizenko et al. 2024). We thoroughly review and analyze preclinical data from the previous decade to examine their use as drug delivery systems (DDS).

### 2.1 Isolation Strategies of EVs

**FIGURE 3:**
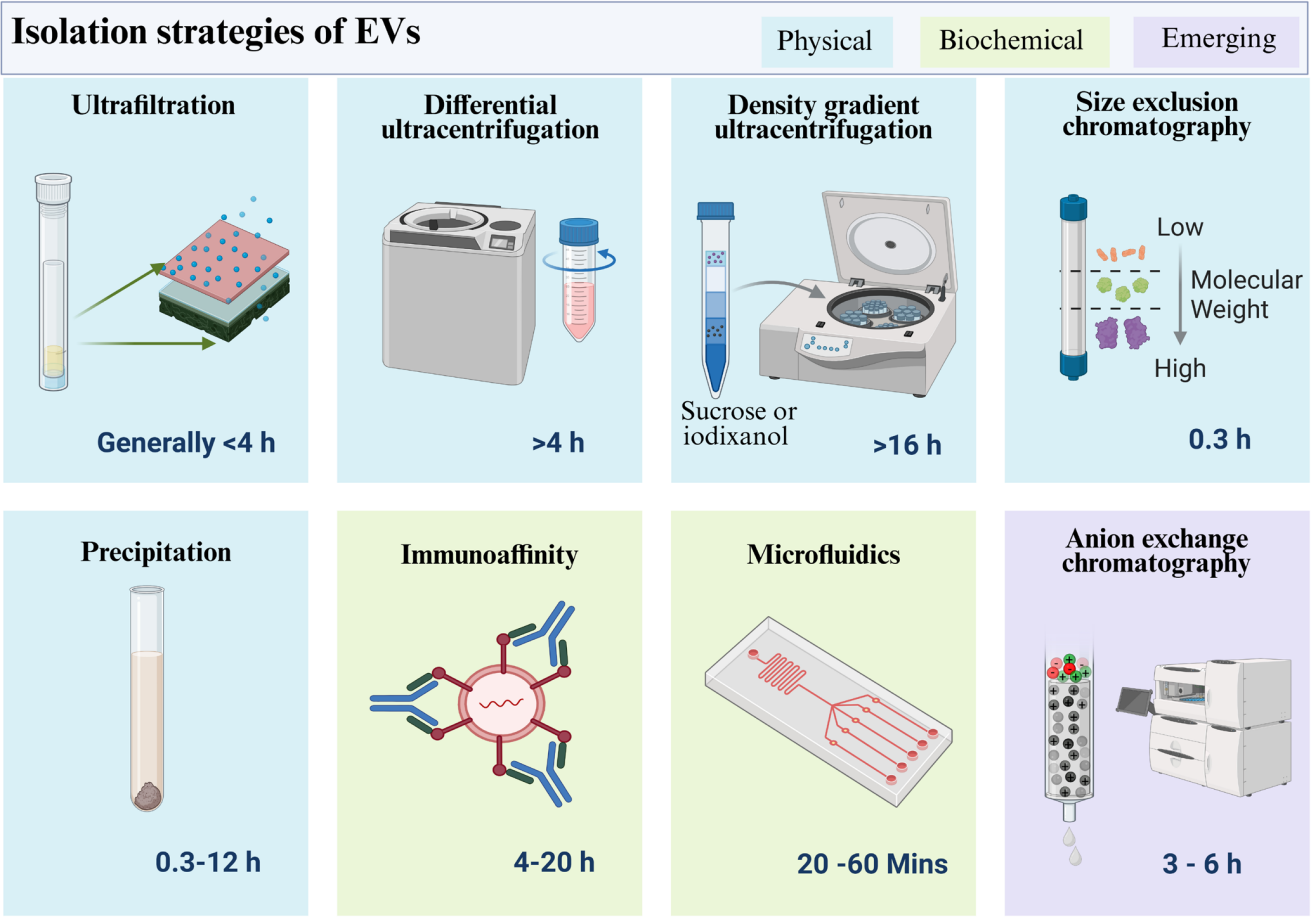
Isolation methods of EVs. EVs are isolated by using various techniques. Physically, EVs are isolated using ultrafiltration, ultracentrifugation, density gradient centrifugation, size exclusion chromatography, and precipitation. Under biochemical strategy, immunoaffinity and microfluidic techniques are commonly used to isolate the vesicles precisely. The anion exchange chromatographic method is an emerging technique. In addition combination of these methods is also in practice.

Since the isolation approach affects the EV population and study outcome, it must be carefully considered for clinical use. EVs can be isolated and purified from bodily fluids and cell culture supernatants utilizing many methods (Figure 3; Admyre et al. 2007; Carnino et al. 2019). EV isolation method selection depends on the fluid (blood, cell culture supernatant, urine, etc.), volume, and required EV purity. As a clinical treatment, isolated EVs must be uncontaminated, especially free of proteins and nucleic acids that could affect clinical administration. Separating EVs from proteins and nucleic acids guarantees that therapeutic vesicles' biological effects are due to EV payloads, not co‐purified impurities. EV purity may not matter for clinical biomarker investigations, depending on the study. Sequencing, ELISA, or nanoscale flow cytometry would focus on quantity rather than purity for biomarker analysis. However, biomarker discovery investigations require excellent purity and EV characterization before validation and clinical applications.

The standard EV isolation method is differential ultracentrifugation. This procedure, known as the “gold standard” for EV separation, involves centrifugation to remove cells and debris from cell culture supernatant (300 g and 2500×*g*), pellet large EVs (10,000×g), and finally small EVs (100,000×*g*/ 200,000×*g*). Literature results suggest that the reproducibility of isolation experiments is highly varied with the parameters such as rotor type (swing bucket vs. fixed angle), sample viscosity, and tube k‐factor. From this process, about 66% of EV preparations utilize high centrifugal forces that may result in aggregated proteins and other impurities. Ultracentrifugation alone cannot extract lipoproteins from biological samples like blood without a gradient or other chromatographic methods. In the density gradient approach, EVs can be separated from contaminating proteins by layering sucrose or iodixanol solutions of increasing concentrations (Théry et al. 2006; Zhang et al. 2020). EVs contained in lipids float higher during ultracentrifugation (200,000×*g* overnight) based on density, separating them from contaminating proteins (Kurian et al. 2021; Schulz‐Siegmund and Aigner 2021; Taylor and Shah 2015). Ultracentrifugation is useful for laboratory research but impractical for clinical usage because of its time‐consuming preparation, equipment requirements, and low throughput scalability.

However, ultrafiltration methods like tangential flow filtration (or sequential) can quickly isolate EVs from vast cell culture supernatants and biological fluids (Hou et al. 2024). Tangential flow filtration allows proteins and liquid to pass through a membrane filter with a molecular weight cut‐off (usually 500 kDa) while keeping EVs in the retentate. This method concentrates large or small liquid quantities while capturing EVs. The approach produces EVs with substantial protein contamination, and the filtering membrane may damage EV integrity. Tangential flow filtering must be utilized with size exclusion chromatography to achieve high purity. Alternatively, sequential filtering isolates protein‐free EVs in three phases. Dead‐end filtering removes cells and detritus first. The sample is then concentrated, and EVs are retained via tangential flow filtration. Final filtering via a track‐edged membrane with increasing pore sizes (50–200 nm) isolates and fractionates EVs by size (Shu et al. 2021; Vergauwen et al. 2017; Yuan et al. 2023).

Size exclusion chromatography efficiently separates particles by size. This is used to decontaminate EVs from complex biological samples' proteins. When biological fluids like blood plasma or serum are processed into a Sepharose‐based size exclusion column, the differential exclusion approach navigates EVs elution at first and proteins to elute in later fractions. Size exclusion chromatography cannot efficiently filter EVs from plasma or serum lipoproteins of equal size. Density gradient ultracentrifugation and size exclusion chromatography are needed to completely remove lipoproteins. Affinity and ion exchange chromatography are other EV purifying methods (Sidhom et al. 2020; Merij et al. 2024). Membrane affinity purification technologies, such as exoEasy spin columns, are used to isolate EVs from the biological samples, but the purity of the isolated EVs may be lower than that of size exclusion chromatography (Masaki et al. 2023; Stranska et al. 2018; Yang et al. 2021). Tim4, a calcium‐sensitive phosphatidylserine binding protein, has optimized affinity‐based approaches. EVs bound to Tim4 can be freed by calcium chelators (Kawakami et al. 2021; Wang, Liu, et al. 2023; Yoshida et al. 2017). Other commonly available immuno‐affinity capture agents are heparin, epithelial cells, tetraspanins, and adhesion molecules (Balaj et al. 2015; Gurunathan et al. 2022; Tauro et al. 2012). However, immuno‐affinity capture agents only purify certain EV populations and can be difficult to extract from the substrate without extreme conditions like low pH. Anion exchange chromatography is the final chromatographic method for scalable and effective EV separation from cell culture supernatant (Koch et al. 2024; Pirolli et al. 2023). Positively charged columns bind negatively charged EVs, which are eluted with increasing salt concentrations. Anion exchange chromatography can separate EVs from 1 L of cell culture‐conditioned media in 2 h with minimal user input. This method for EV isolation is scalable and fast, suggesting it could enable EV therapy (Pirolli et al. 2024; Silva et al. 2023).

EVs are often isolated from clinical biological samples by precipitation with commercial reagents. EVs can be pelleted by centrifugation at lower speeds without ultracentrifugation by precipitating them with polyethylene glycol (PEG) or commercial reagents like exoquick86 (Ding et al. 2018; Ludwig et al. 2018). EVs can be captured from tiny biological fluids or preconcentrated biological fluids/cell culture supernatants. Although user‐friendly and suitable for many biological samples, precipitation can pellet proteins and lipoproteins, lowering EV purity. A pure EV preparation may require a second purification step after precipitation. Precipitation reagents in EV preparations can also alter recipient cell survival and biological activity.

Finally, microfluidic devices can isolate and analyze EVs from small clinical samples and may be beneficial for liquid biopsy disease detection. Immunocapture microfluidic devices use tumor‐specific antigens or other markers (Kwon et al. 2025; Park et al. 2025). HER2 and PSA‐positive tumor‐derived EVs have been collected on chips using nano‐shearing fluid flow (Mun et al. 2024). EGFR wild type or EGFR v III EVs may be identified and measured from glioblastoma plasma. Eluted EVs from a chip were employed for more in‐depth EGFR v III EV RNA sequencing. An alternate chip device used EpCAM aptamers to capture EVs and electro‐oxidation of metal NPs to detect EpCAM and PSMA epitopes (Amrollahi et al. 2019; Salmond and Williams 2021; Sun et al. 2023; Zhu et al. 2023). Electrochemical peaks from metal particle oxidation can be utilized to quantify collected EVs (Zhou et al. 2016). Microfluidic devices with particular size thresholds catch tumor‐derived microvesicles. EVs pass through the microfluidic chip and are eluted from size‐dependent ports for downstream processing. Table 1 summarizes the advantages and disadvantages of typical EV isolation methods.

**TABLE 1:** Advantages and disadvantages of currently practiced EV isolation techniques.

Isolation methods	Principle	Used for	Advantages	Limitations	References
Ultracentrifugation	Isolation of EVs based on size, shape, and density using high‐speed centrifugation.	Isolation of large EVs from small EVs.	Well‐defined and common approach technique. Especially used to separate and isolate large EVs from small EVs.	Time‐taking method. Substantial required equipment needs. Contaminating proteins and lipoproteins remain unremoved.	(Abramowicz et al. 2016; Cvjetkovic et al. 2014; Livshits et al. 2016; Momen‐Heravi et al. 2013; Théry et al. 2006)
Gradient	Density gradient centrifugation utilizes the disparities in density between extracellular vesicles and other molecules or particles within a sample with the help of sucrose or iodixanol.	Purification of EVs from the contaminants.	Isolate the EVs from protein contaminants and some lipoproteins.	Time‐consuming method. Special equipment is needed. Additional purification procedures may be required for the complete elimination of lipoprotein.	(Greening et al. 2015; Salih et al. 2014; Tauro et al. 2012; Taylor and Shah 2015)
Ultrafiltration	Utilizes porous membranes with particular pore sizes to retain EVs while allowing smaller molecules and particles to pass through.	Isolating EVs from large sample volumes.	Expeditious extraction of EVs from substantial quantities.	Protein and lipoprotein contamination were not removed.	(Konoshenko et al. 2018; Lobb et al. 2015; Salih et al. 2014; Taylor and Shah 2015)
Size exclusion chromatography	Size‐based separation using porous matrices.	Used to isolate and purify small EVs and Exosomes.	Rapid separation of EVs from minimal biological samples or from larger volumes that have undergone preconcentration.	Low scalability for high throughput. Additional purification steps are required for the removal of lipoproteins.	(Abramowicz et al. 2016; Gámez‐Valero et al. 2016; Konoshenko et al. 2018; Salih et al. 2014; Taylor et al. 2011; Taylor and Shah 2015)
Affinity	Relies on specific binding to specific surface markers via immobilized ligands such as antibodies on a stationary phase.	Specific EV subpopulations (e.g., CD63+, CD9+ exosomes).	Rapid isolation procedure.	Specifically isolates particular EV populations. Challenging to extract from beads without damage.	(Konoshenko et al. 2018; Salih et al. 2014; Tauro et al. 2012; Taylor and Shah 2015)
Anion exchange chromatography	Anion exchange chromatography separates extracellular vesicles (EVs) by utilizing their negative surface charge, adhering them to a positively charged resin, and subsequently eluting them with a high‐salt buffer.	Used to isolate Exosomes and microvesicles from cell culture supernatant and serum.	Rapid extraction of EVs from large volumes of cell culture media. Preliminary evidence indicates elevated purity.	The necessity for additional purification processes must be assessed.	(Chen et al. 2018; Koch et al. 2024; Konoshenko et al. 2018; Malvicini et al. 2023)
Precipitation	Utilizes the concept of altering EV solubility and dispersibility in aqueous solutions, frequently employing polymers such as polyethylene glycol (PEG) or charge‐based interactions to precipitate EVs and segregate them from other cellular constituents.	Used to isolate exosomes and microvesicles.	The rapid isolation method from biological samples. High‐throughput scalability.	Preconcentration is needed for the higher volume of samples. Additional purification steps are often required to remove contaminating proteins and lipoproteins.	(Andreu et al. 2016; Gámez‐Valero et al. 2016; Konoshenko et al. 2018)
Microfluidic chips	The miniaturized channels and chambers are used to manipulate and separate the EVs according to their physical features, such as size and density, or their affinity for certain surface markers, so enabling rapid and sensitive assessment.	Isolation of exosomes from a small volume of samples.	Rapid processes for isolation of EVs from small amounts of biological samples. High‐throughput scalability.	Engineering and fabrication of chips are required, not easily available in the commercial market.	(He et al. 2014; Kanwar et al. 2014; Ko et al. 2016; Santana et al. 2014)

### 2.2 Extracellular Vesicles as Drug Delivery Systems

Several research works have evidenced that EVs are excellent candidates for various therapeutic and diagnostic applications. The dynamic protein sources in the outer layer channel these EVs to specific targets. So, the encapsulated materials are actively delivered to the specific target sites. Table 2 lists the numerous sources and cell types that are widely utilized to isolate EVs, as well as their applications. The therapeutic and diagnostic payloads contained within the EVs are detailed in Table 3.

**TABLE 2:** Commonly used cell types to isolate EVs and their applications.

Cell type	Primary cells/cell lines/source	EVs application area	Significance	References
Mesenchymal stem cells (MSCs)	Bone marrow‐derived MSCs (BM‐MSCs)	Cardiac therapy, immune modulation, tissue repair	Widely used because of its regenerative and immune‐modulatory properties.	(Aguiar Koga et al. 2023; Bertolino et al. 2022; Chen, Qu, et al. 2021; Ding et al. 2024; Lelek and Zuba‐Surma 2020; Matsuzaka and Yashiro 2022; Mutlu et al. 2015; Ulpiano et al. 2023; Um et al. 2020; Wang, Xu, et al. 2023; Yang, Sun, and Yan 2024; Zhao et al. 2024)
	Adipose tissue‐derived MSCs (AD‐MSCs)	Arthritis, wound healing, and skin repair		
	Umbilical cord‐derived MSCs (UC‐MSCs)	Immunotherapy, anti‐fibrosis, neuroprotection		
	Placenta‐derived MSCs (P‐MSCs)	Inflammation, autoimmune disorders		
	Amniotic fluid‐derived MSCs (AF‐MSCs)	Prenatal therapy, neurodevelopmental disorders		
	Dental pulp‐derived MSCs (DP‐MSCs)	Bone regeneration, Neuroprotection		
	Induced pluripotent stem cell‐derived MSCs (iPSC‐MSCs)	Cardiovascular repair, personalized therapy		
	Synovial membrane MSCs (SM‐MSCs)	Osteoarthritis, joint repair		
	Endometrium‐derived MSCs (eMSCs)	Wound healing, uterine regeneration		
	Menstrual blood‐derived MSCs (MenSCs)	Neuroprotection, endometrial repair		
Immune cells	Dendritic cells (DCs)	Cancer immunotherapy, vaccines	EVs carry immune signals and MHC molecules.	(Gargiulo et al. 2020; He et al. 2024; Kowal and Tkach 2019; Lin et al. 2022; Lou et al. 2022; Putthanbut et al. 2024; Shefler et al. 2021; Wang et al. 2021; Wen et al. 2017; Zhang, Liu, et al. 2022)
	T cells (CD4+, CD8+)	Immunomodulation, anti‐viral application, EV‐based cytotoxicity		
	B cells	Antigen presentation, autoimmune disease models		
	Macrophages	Inflammation, Infection models, Wound healing		
	Natural killer (NK) cells	Cancer immunotherapy, cytotoxic EV studies		
	Neutrophils	Inflammatory modulation, anti‐microbial activity		
	Monocytes	Atherosclerosis, chronic inflammation		
	Regulatory T cells (TRegs)	Immune tolerance, suppressing autoimmunity		
	Mast cells	Neurodegenerative disease models, brain injury response		
Cancer cells	Breast cancer (MCF‐7, MDA‐MB‐231, BT‐474)	Diagnostic biomarkers, drug resistance studies, immune modulation	Carries parent cell tumor antigens and reflects tumor microenvironment	(Acevedo‐Sánchez et al. 2021; Chang et al. 2021; Chen, Jin, and Wu 2021; Qi et al. 2021; Saviana et al. 2021; St‐Denis‐Bissonnette et al. 2022; Strum et al. 2024)
	Glioblastoma (U87, U251, LN229)	Brain‐targeted drug delivery, tumor progression		
	Lung cancer (A549, H1299, H460)	Anti‐metastatic applications, immune evasion		
	Colorectal cancer (HCT116, SW480, HT‐29)	Diagnostic applications, anti‐metastasis, and cell signaling		
	Prostate cancer (PC3, DU145, LNCaP)	Biomarker development, EV‐based liquid biopsy		
	Pancreatic cancer (PANC‐1, AsPC‐1, BxPC‐3)	Early diagnosis, drug resistance models		
	Ovarian cancer (SKOV3, OVCAR3)	Anti‐metastasis, EV‐based biomarker discovery		
	Melanoma (B16‐F10, A375)	Vaccine development, Immunotherapy		
	Leukemia (K562, HL‐60, Jurkat)	Drug resistance, hematologic malignancy monitoring		
	Hepatocellular carcinoma (HCC; HepG2, Huh7)	Anti‐angiogenesis, liver cancer biomarkers for diagnostic applications		
	Cervical cancer (HeLa, SiHa)	Diagnosis, oncoviral studies		
Epithelial cells	Renal epithelial cells (HK‐2, primary renal epithelial cells)	Diagnostic biomarkers for kidney injury, urinary diagnostics	Handling the cell is simple, easy to culture, and enhanced EV yield	(Chen et al. 2022; Cui et al. 2024; Pirisinu 2023; Xue and Mi 2024)
	Mammary epithelial cells (MCF10A, HMEC)	Breast cancer research, intercellular signaling		
	Bronchial epithelial cells (BEAS‐2B, primary bronchial cells)	Lung inflammation, asthma, COPD, COVID‐19		
	Intestinal epithelial cells (Caco‐2, HT‐29, enterocytes)	Gut immunity, host–microbe communication		
	Corneal epithelial cells (HCE‐T, primary corneal epithelial cells)	Immune modulation, ocular surface healing		
	Prostate epithelial cells (RWPE‐1, PrEC)	Cancer diagnostics, prostate health		

	Cervical epithelial cells (HeLa, primary cervical cells)	Cancer biomarker discovery, HPV infection studies		
	Salivary gland epithelial cells (HSG, primary SGECs)	Saliva‐based diagnostics, autoimmune research		
	Skin keratinocytes (HaCaT, NHEK)	Wound healing, skin inflammation		
Liver epithelial cells (HepG2, primary hepatocytes)	Liver disease modeling, drug toxicity			
Neural cells	Neurons (primary cortical neurons, SH‐SY5Y)	Neurodegenerative disease modeling	Carry synaptic and neuronal proteins	(Li, Zhu, et al. 2023; Li and Fang 2023; Marangon et al. 2023; Zappulli et al. 2016; Zhu et al. 2024)
	Astrocytes (Primary astrocytes, U373)	Neuronal protection, neuroinflammation		
	Microglia (BV2, primary microglia)	Neuroinflammation, brain injury		
	Oligodendrocytes (OLN‐93, primary oligodendrocytes)	Myelin support		
	Neural stem cells (hNSCs, ReNcell VM)	Brain development, regenerative medicine		
	Schwann cells (primary Schwann cells)	Peripheral nerve regeneration		
	Retinal ganglion cells (primary Schwann cells)	Glaucoma, optic nerve injury		
Endothelial cells	Human umbilical vein endothelial cells (HUVECs)	Angiogenesis, inflammation, atherosclerosis	Release EVs in response to stress or inflammatory signals	(Deng et al. 2024; Liu et al. 2023; Piryani et al. 2024; Terriaca et al. 2021; Villata et al. 2020; Weksler et al. 2013; Yang et al. 2023)
	Brain microvascular endothelial cells (hCMEC/D3, primary BMECs)	Blood–brain barrier integrity, neurovascular research		
	Coronary artery endothelial cells (HCAEC)	Cardiovascular disease, ischemia–reperfusion		
	Aortic endothelial cells (HAEC)	Atherosclerosis, inflammation research		
	Pulmonary artery endothelial cells (HPAEC)	Pulmonary hypertension, lung injury		
	Lymphatic endothelial cells (HDLEC)	Lymphangiogenesis, tumor metastasis		
	Microvascular endothelial cells (HMEC‐1, dermal MVECs)	Microcirculation, skin repair		
Platelets	Resting platelets (healthy donor blood)	Hemostasis regulation	Release platelet‐derived microvesicles (PMVs)	(Anitua et al. 2023; Chaudhary et al. 2023; Das et al. 2024; Fan et al. 2024)
	Activated platelets (thrombin/ADP/collagen‐stimulated platelets)	Coagulation, inflammation, and cancer		
	Stored platelets (blood bank platelets)	Transfusion quality control		
	Platelet‐rich plasma (PRP from blood)	Regenerative therapy		
	Megakaryocytes (MEG‐01, primary cells)	Platelet biogenesis		
	Platelets under shear stress (shear flow‐exposed platelets)	Thrombosis, atherosclerosis		
Fibroblasts	NHDFs (primary dermal fibroblasts)	Wound healing, anti‐aging	Often used for baseline EV studies	(Hua et al. 2021; Laurent et al. 2021; Mehta, Kadoya, et al. 2023; Prieto‐Vila et al. 2024; Sui et al. 2023; Xie et al. 2025; Zhang et al. 2024)
	HFFs (HFF‐1, BJ fibroblasts)	Skin repair, cosmetics		
	MEFs (primary MEFs)	Developmental biology		
	CAFs (tumor‐associated fibroblasts)	Cancer progression		
	Gingival fibroblasts (primary gingival fibroblasts)	Oral regeneration		
	Lung fibroblasts (MRC‐5, WI‐38)	Pulmonary fibrosis		
	Cardiac fibroblasts	Heart failure therapy		
	Endometrial fibroblasts (primary endometrial stromal cells)	Fertility research		

**TABLE 3:** List of therapeutic and diagnostic payloads loaded into EVs.

Payload type	Examples	Loaded into EV type	Purpose/application	References
*Therapeutic applications*				
Small‐molecule drugs and chemotherapeutics	Paclitaxel, doxorubicin, curcumin, cisplatin, gemcitabine, methotrexate, temozolomide, imatinib, resveratrol, rapamycin	Exosomes and microvesicles	Targeted cancer therapy, reduced systemic toxicity, tissue injury, and antimalarial.	(Akbari et al. 2023; Bellavia et al. 2017; Chew et al. 2025; Kim, Kim, et al. 2021; Li, Zhang, et al. 2022; Liu and Zhang 2024; Wang et al. 2022; Wang et al. 2024; Yang, Wang, et al. 2024; Zhao et al. 2021; Zheng et al. 2023)
Nucleic acids	siRNA, miRNA, shRNA, mRNA, circRNA, DNA plasmid, aptamer, GuideRNA, shRNA	Exosomes, microvesicles, tumor EVs, and engineered exosomes	Gene regulation, silencing, gene expression, targeted therapy, diagnostic imaging, epigenetic regulation, and cancer signaling	(Ahmed et al. 2024; Akbari et al. 2023; Ebrahimi et al. 2024; Huang et al. 2023; Lin et al. 2020; Silva and Melo 2015)
Proteins and peptides	Catalase, cytokines, tumor antigens, superoxide dismutase (SOD), GFP (green fluorescent protein), interleukin‐10 (IL‐10), heat shock proteins (HSP70, HSP90), CD47, BCL‐2 peptide mimetics, cytotoxic T‐cell receptors	Exosomes, engineered exosomes, and tumor EVs	Antioxidant, immunotherapy, neuroprotection in Parkinson's, stroke, and inflammation.	(Abdal Dayem et al. 2024; Ferrantelli et al. 2022; Huang et al. 2023; Li et al. 2025; Tang et al. 2020; Silva et al. 2021; Taghikhani et al. 2020; Xu et al. 2020)
CRISPR/Cas systems	Cas9/sgRNA complexes, Cas12a, Cas13a	Exosomes, engineered exosomes (HEK293, macrophage, MSC), and EV‐mimetic nanovesicles	Targeted gene editing (CRISPR), genome editing of mutated genes (e.g., PCSK9, CCR5), epigenetic silencing of gene expression, and upregulating genes like P53 or anti‐inflammatory genes.	(Li, Zhang, et al. 2023; McAndrews et al. 2021; Song et al. 2024; Yao et al. 2021; Ye et al. 2020)
Immunomodulators	IL‐10, tumor necrosis factor‐α (TNF‐α) inhibitors, transforming growth factor‐β (TGF‐β), IL‐1 receptor antagonist (IL‐1Ra), CTLA‐4, galectin‐9, IDO (indoleamine‐2,3‐dioxygenase)	Exosomes and engineered exosomes	Modulating immune response in autoimmune/inflammatory diseases, immune tolerance, allograft acceptance, rheumatoid arthritis, psoriasis, and tumor immune evasion studies.	(Cao et al. 2024; Luo et al. 2025; Pingquan et al. 2023; Tang et al. 2020; Taghikhani et al. 2020)
Antibiotics/antivirals	Ciprofloxacin, acyclovir, gentamicin, vancomycin, amphotericin B, rifampicin, antiviral siRNA, tenofovir	Exosomes from various sources.	Targeted infection control (HIV, HSV, TB), bacterial infections, and anti‐viral applications.	(Davari et al. 2023; Mondal et al. 2024; Song et al. 2022; Tun‐Yhong et al. 2017; Zhang, Dai, et al. 2022)
Enzymes	β‐Gluco‐cerebrosidase, glutathione peroxidase (GPx), neuraminidase (sialidase), DNase I, asparaginase, and alkaline phosphatase	Exosomes and engineered exosomes	Parkinson's, lysosomal storage disorders, anti‐inflammatory, neuroprotection, Gaucher's disease, cancer therapy, anti‐thrombosis, cystic fibrosis, autoimmune disease, and cancer immunotherapy	(Alli 2021; Bonucci et al. 1992; Delaveris et al. 2023; Du, Chen, et al. 2023; Iraci et al. 2017; Kim et al. 1999)
Nanoparticles	Gold NPs, lipid‐polymer hybrid NPs, silica nanoparticles, and nanoceria (CeO_2_ NPs)	Engineered exosomes	Theranostics combined therapy, photothermal therapy, co‐delivery of drugs and genes, antioxidant therapy, gene delivery, ROS scavenging, and neuroprotection	(Ayed et al. 2019; Bader et al. 2025; Gao et al. 2023; Ivanova et al. 2025; Tan et al. 2017)
*Diagnostic applications*				
Tumor‐derived miRNAs	miR‐21, miR‐155, miR‐1246, miR‐210, miR‐19b, miR‐200c, and miR‐10b	Exosomes	Biomarkers for early cancer detection, tumor progression, hypoxia marker, tumor angiogenesis, EMT biomarker, invasion, and metastasis predictor.	(Kim, Park, et al. 2021; Kim, Kim, et al. 2024; Molina‐Pelayo et al. 2025; Zhou, Pan, et al. 2024)
Oncoproteins	EGFRvIII, HER2, mutant p53, EpCAM, survivin, and PSA (prostate‐specific antigen)	Exosomes	Tumor‐specific signatures in liquid biopsy, oncogenic driver mutation, early‐stage detection, epithelial tumor marker, EV trafficking—tumor cell migration marker, prognostic biomarker, and tumor progression monitoring.	(Bregola et al. 2023; Frawley and Piskareva 2020; González et al. 2024; Huang, Rao, et al. 2022; Kato et al. 2021; Liao et al. 2024; Oey et al. 2021; Pavlakis and Stiewe 2020; Verma et al. 2015)
Lipid profiles	Phosphatidylserine, ceramides, sphingomyelin	All EVs	EV identification, staging of cancer	(Burrello et al. 2020; Chang et al. 2021; Dinkins et al. 2017; Perez et al. 2023; Izadpanah et al. 2018)
Fluorescent dyes	DiI (DiR), PKH26, CFSE	Exosomes	In vivo/ex vivo imaging and biodistribution tracking	(Arifin et al. 2022; Cha et al. 2023; Dehghani and Gaborski 2020; González et al. 2021; Panagopoulou et al. 2020)
Magnetic nanoparticles	SPIONs, gold‐iron hybrid NPs, gadolinium, and MnFe_2_O_4_ nanoparticles.	Engineered exosomes	MRI‐based EV tracking, dual‐mode imaging (MRI + CT/PAI), image‐guided delivery and tracking	(Hamzah et al. 2022; Rayamajhi et al. 2020; Sanavio and Stellacci 2017; Yang et al. 2022a, 2022b)
Radiotracers	^64^Cu, ^99^mTc, ^125^I	Exosomes	PET/SPECT imaging of EV biodistribution	(Arifin et al. 2022; N'Diaye et al. 2022; Son et al. 2020; Yang, Guo, et al. 2024; Yerneni et al. 2022)

**FIGURE 4:**
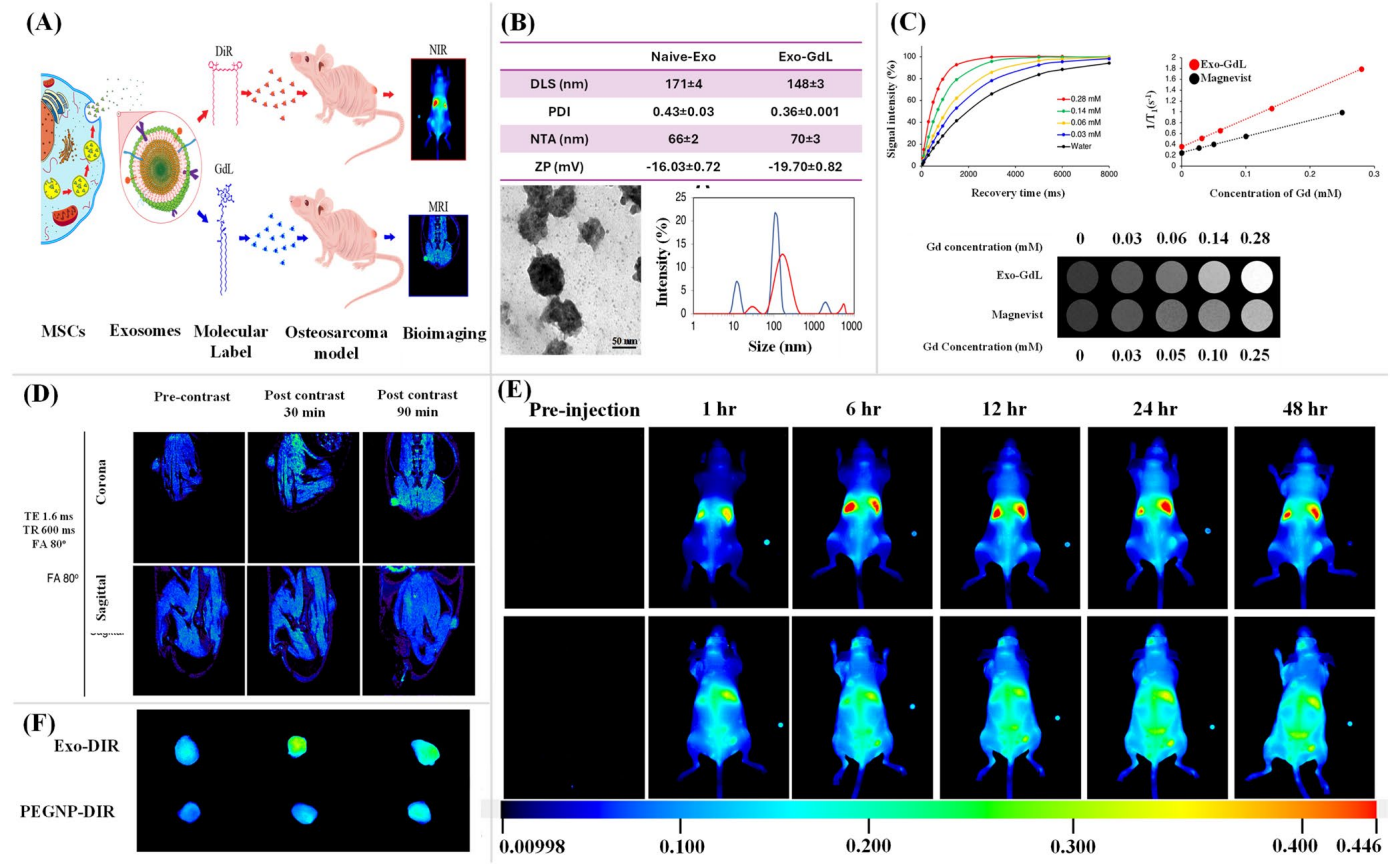
Gadolinium and near‐infrared‐labeled human umbilical cord mesenchymal stromal cell exosomes development and the assessment of their tumor targeting efficacy in tumor‐bearing mice. (A) Schematic representation of the overall study. (B) DLS, Zeta, NTA, and TEM Characterization of Exosomes and Exo‐GdL showed the accessible structural characteristics for active drug delivery. (C) Magnetic properties of Exo‐GdL. The Exo‐GdL showed enhanced contrast when compared to Magnevist. (D) The magnetic resonance imaging of tumor‐bearing mice to assess the Exo‐GdL distribution. (E) Biodistribution of NIR dye‐labeled exosomes in ectopic osteosarcoma mice model. (F). Exo‐DiR accumulation in mouse K7M2 osteosarcoma tumors. The in vivo studies demonstrated that the exosomes derived from hMSC showed enhanced targeting ability. (Reprinted with permission from Abello et al. (2019). Copyright 2019 Ivyspring International Publisher).

Abello et al. have undertaken a comprehensive investigation into the use of labeled exosomes as potential diagnostic and therapeutic tools. The authors have utilized gadolinium for MRI and near‐infrared (NIR) fluorescence imaging to track the biodistribution of exosomes derived from human umbilical cord mesenchymal stromal cells (hUC‐MSCs) in tumor‐bearing mice (Abello et al. 2019). This dual‐labeling approach provides a robust strategy to monitor exosome trafficking with high sensitivity and resolution (Figure 4A). The exosomes were isolated from hUC‐MSCs and characterized by dynamic light scattering (DLS), nanoparticle tracking analysis (NTA), and transmission electron microscopy (TEM) and confirmed the presence of protein markers using dot blot and western blot. After that, the gadolinium lipid (GdL) was loaded into the exosomes. The characterization studies showed the naive exosomes exhibited a hydrodynamic size of 171 ± 42 nm with a polydispersity index (PDI) of 0.43 ± 0.03 and zeta potential at −16.03 ± 0.72 mV, indicating good colloidal stability, suggesting a surface composition favorable for cellular uptake. Meanwhile, the GdL‐exosomes exhibited a hydrodynamic size of 148 ± 3 nm and a zeta potential of −19.70 ± 0.82 mV (Figure 4B). The longitudinal relaxivity (r1) of Exo‐GdL was assessed with a 14.1 T MRI system, resulting in an r1 value of 5.1 mM^−1^ s^−1^, in contrast to 2.9 mM^−1^ s^−1^ for Magnevist (clinical agent). T1‐weighted MRI images validated the superior contrast of Exo‐GdL at equivalent Gd concentrations relative to Magnevist, indicating its potential as a highly sensitive MRI contrast agent (Figure 4C). These findings highlight the efficacy of exosome‐based contrast agents in enhancing imaging sensitivity and resolution. In vivo MRI studies in osteosarcoma‐bearing mice revealed that Exo‐GdL accumulated at the tumor site in a time‐dependent manner, with peak enhancement at 30 and 90 min after injection (Figure 4D). ICP‐MS analysis revealed 18% of injected Exo‐GdL accumulated in the tumor after 24 h, outperforming conventional liposomes and NPs due to the natural homing ability of hUC‐MSC‐derived exosomes and the EPR effect. Tumor targeting was twice as effective as Magnevist, and near‐infrared imaging confirmed longer circulation and better targeting than PEGylated NPs, indicating potential for targeted cancer diagnostics and therapy (Figure 4E,F). The study highlights the ability of exosomes to preferentially accumulate in tumor tissues, emphasizing their potential as targeted drug delivery vehicles. Additionally, the findings provide valuable insights into the pharmacokinetics and tumor microenvironment interactions of exosomes. This work represents a significant advancement in the application of nanomedicine for cancer diagnostics and therapy, offering a promising platform for further exploration in preclinical and clinical settings. However, future studies focusing on the mechanisms of tumor targeting and potential immunological responses will be crucial for clinical translation (Abello et al. 2019).

**FIGURE 5:**
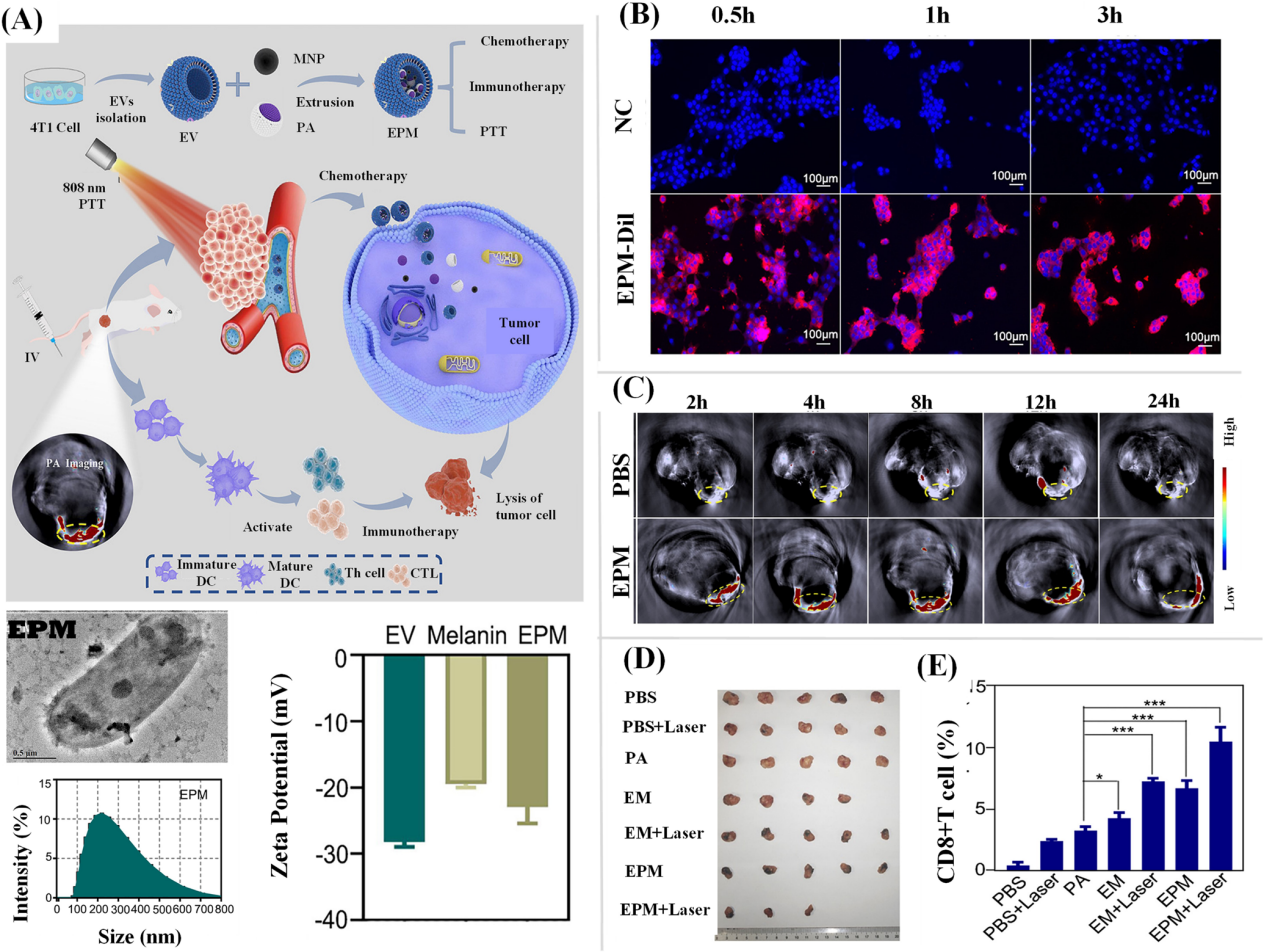
The drug delivery system developed from tumor‐derived EVs with the combination of chemo, photothermal, and immune therapy. (A) An overview of EVs development strategy and the characterization of EVs loaded with paclitaxel and melanin (EPM). The characterization studies showed the efficient loading of drugs, and also these vesicle employs features that are capable of targeting and delivering the cargo. (B) In vitro cellular uptake assessment by flowcytometry and laser scanning confocal microscopy. The enhanced fluorescent intensity in EPM‐treated 4T1 cells revealed that the system actively internalized into the cells. (C) Time‐dependent photoacoustic images of orthotopic breast cancer mice model treated with EPM showed a higher accumulation of EVs in the target site over the periods of 2, 8, and 12 h. (D) Assessment of tumor size in time‐dependent manner. (E) Assessment of the immune effect of EPM in CD8 + T cells (Reprinted with permission from Bi et al. (2024). Copyright 2024 Elsevier Ltd).

Several researchers show high interest in tumor‐derived EVs (TEVs), which have remarkable functional properties and pave the way to target the cancer cells to deliver their cargo. Recently, Bi and his research group derived the TEVs from mouse breast cancer cells and developed a multifunctional drug delivery platform for effective cancer treatment (Bi et al. 2024). This study focused on developing the nano platform loaded with melanin and paclitaxel albumin. The work employs EVs as a drug delivery system, leveraging their inherent tumor‐targeting ability, biocompatibility, and low immunogenicity to ensure accurate administration of therapeutic drugs while reducing off‐target effects. The study combines chemotherapy, photothermal therapy, and immunotherapy into a cohesive system, exhibiting a strong synergistic impact that significantly inhibits tumor growth and metastasis. The combinatorial chemo‐photothermal therapy facilitates effective tumor ablation, while the use of immunomodulatory drugs stimulates systemic anti‐tumor immunity, thereby diminishing the risk of recurrence. EVs were produced and isolated from 4T1 mouse breast cancer cells. The EVs loaded with paclitaxel albumin and melanin (EPM) were formulated by co‐extruding EVs with Paclitaxel Albumin (PA) and melanin to obtain a spherical‐shaped vesicle with an average size of 113.9 nm. The other characterization studies showed the efficient loading of drugs. Melanin loading and stability in the biological medium were confirmed by a zeta potential drop from −24.8 to −28.5 mV. HPLC showed 91.51% paclitaxel encapsulation and 31.73% melanin encapsulation, demonstrating the system's drug‐loading effectiveness (Figure 5A). The in vitro cellular uptake by fluorescent labeling showed that EPM is rapidly internalized by breast cancer cells (4T1) with an uptake rate of 99.1% within 3 h. The apoptosis experiments demonstrated that EPM triggered 71.6% apoptosis, markedly exceeding that of free PA. Notably, near‐infrared laser irradiation elevated the apoptotic rate to 87.7%, illustrating the significant synergistic effect of chemotherapy and photothermal therapy (Figure 5B). The in vivo studies were undertaken using an orthotopic breast cancer mouse model to evaluate the therapeutic efficacy of EPM in combination with laser‐induced photothermal therapy. The results revealed increased accumulation of EVs at the tumor site, extended circulation time, and greater treatment efficacy relative to traditional monotherapies (Figure 5C,D). The interesting aspect of this study is the demonstration of immune system activation by EPM through flow cytometry. These results showed that EPM promoted dendritic cells (DCs) by 50.7%, increasing CD80+ and CD86+ markers, which are essential for antigen presentation. EPM + laser increased CD8+ cytotoxic T‐cell infiltration in the tumor microenvironment by 11.2%, supporting this immunological response (Figure 5E). The study notably addresses problems such as medication resistance and significant systemic toxicity, emphasizing the safety and efficacy of the combination method. This research highlights the promise of tumor‐derived EVs as advanced nanocarriers and creates a flexible framework for individualized cancer treatment. This work signifies substantial progress in cancer nanomedicine, facilitating the development of more targeted and varied therapy approaches. This approach not only optimizes drug delivery and therapeutic response but also represents a significant advancement in personalized cancer treatment, paving the path for more effective and less toxic combination therapies (Bi et al. 2024).

**FIGURE 6:**
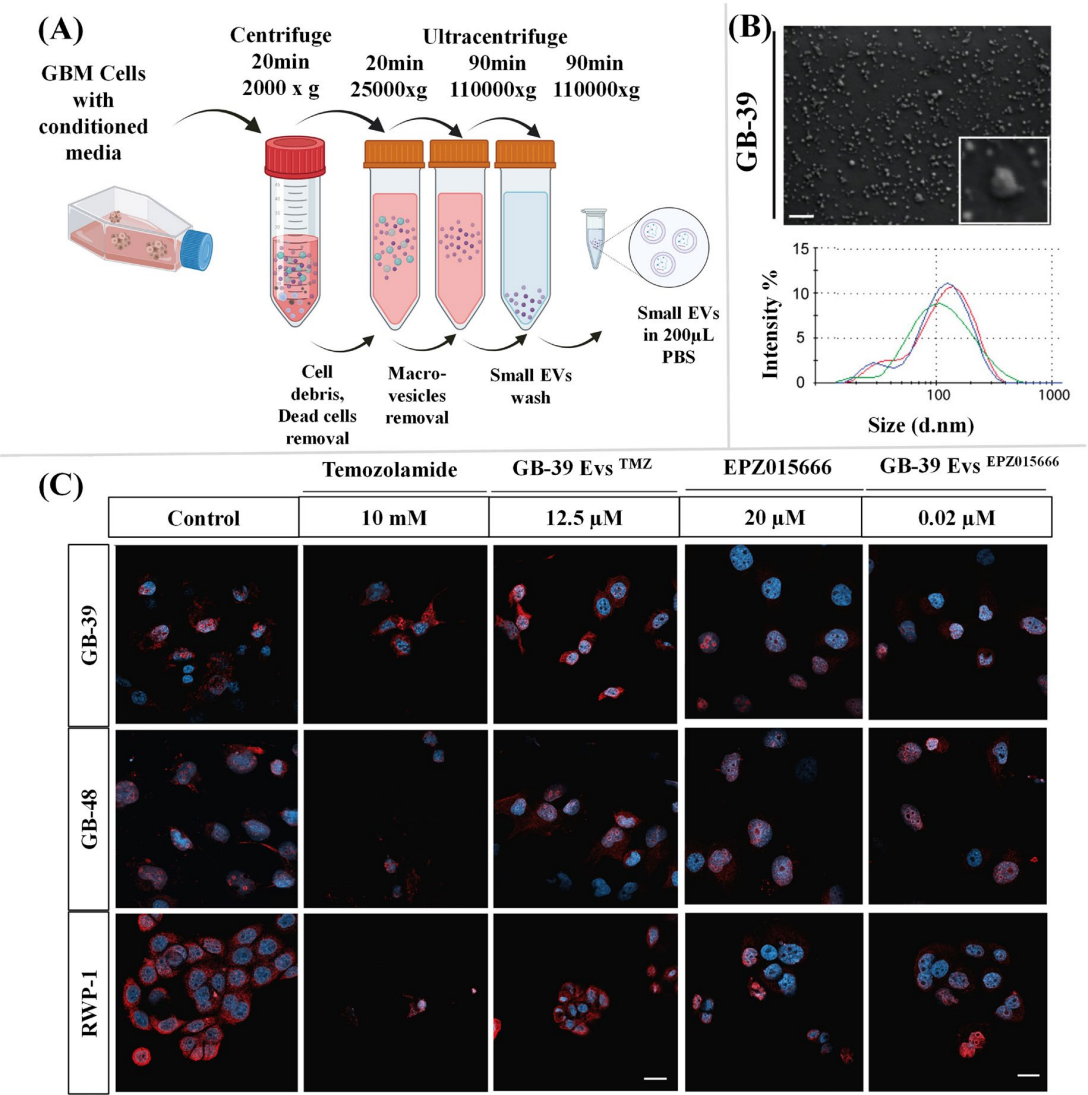
Glioblastoma‐derived small extracellular vesicles (GdMEVs) for glioma treatment. (A) Isolation of small EVs by the ultracentrifugation method. (B) Characterization of GdMEVs through FE‐SEM and DLS. (C) Cell proliferation assessment was undertaken with free drugs Tmozolamide and EPZ015666 inhibitor, along with those compounded loaded EVs. The EVs loaded with EPM showed an efficient proliferation inhibitory effect (Reprinted with permission from Araujo‐Abad et al. (2023). Copyright 2023 MDPI).

Araujo‐Abad and his research group derived EVs from glioblastoma and studied their efficacy as NPs for glioma treatment (Araujo‐Abad et al. 2023). This study provides an innovative perspective on leveraging glioblastoma‐derived small EVs (GdMEVs) as therapeutic agents for glioma treatment. By exploring the dual role of small EVs (sEVs) as both mediators of glioblastoma progression and potential therapeutic carriers, the study accentuates their unique biological characteristics, including intrinsic tumor tropism, the ability to cross the blood–brain barrier, and their biocompatibility (Araujo‐Abad et al. 2023). The authors feature the role of GdMEVs in transporting functional biomolecules, such as proteins, lipids, and nucleic acids, to modify the tumor microenvironment. Furthermore, the paper explores engineering approaches to load therapeutic cargo, such as small interfering RNAs (siRNAs), microRNAs, or chemotherapeutics, into these sEVs to target glioblastoma cells effectively. This strategy represents a promising avenue for precision medicine, addressing the challenges of drug delivery in glioblastoma. However, the authors also acknowledged the hurdles in standardizing sEV isolation, large‐scale production, and minimizing potential off‐target effects (Figure 6). Overall, this study offers compelling evidence for the therapeutic potential of GdMEVs and provides a foundation for future research into nanoparticle‐based strategies for glioma therapy (Araujo‐Abad et al. 2023).

## 3 Liposome Nanoparticles

Surface‐coated liposomes are synthetically tailored drug delivery systems that have garnered considerable interest in both research and clinical applications due to their ability to improve the pharmacokinetics and pharmacodynamics of various therapeutic agents. These liposomes are typically modified on the surface with various molecules to enhance their stability, targeting capability, and circulation time (Chen et al. 2023; Eugster et al. 2024; Gunasekaran et al. 2023; Lee and Thompson 2017; Nsairat et al. 2022). Considering the advantages of the liposome in theragnostic applications, some modifications in the liposome system are needed to enhance the targeting ability, stability, and efficient drug delivery.

Conventional liposome NPs are composed of a phospholipid bilayer and cholesterol, which is uptaken by the Reticuloendothelial system (RES). But these particles have a short half‐life in the blood circulation (Akbarzadeh et al. 2013; Berlin Grace and Viswanathan 2017; Bozzuto and Molinari 2015). Liposomes exhibit their actions in two different modes, such as passive targeting and active targeting. Passive targeting utilizes the enhanced permeability and retention (EPR) effect, a distinctive feature of tumor and inflamed tissues (Haley and Frenkel 2008). The vasculature of diseased tissues is permeable; therefore, the liposomes are more easily accumulated in these tissues compared with normal tissues. This permits the enhanced local concentration of drugs in the tumor site while reducing the drug's exposure to the rest of the body and limiting any associated adverse side effects (Chehelgerdi et al. 2023). Liposomes could aggregate specifically in tumor locations, which is highly beneficial in cancer therapy and diagnostic applications, and the cargo efficiently delivered by this aggregation results in efficient therapeutic activity by targeting the molecular pathways (Berlin Grace et al. 2017; Gunasekaran et al. 2023; Berlin Grace et al. 2025; Viswanathan et al. 2019; Viswanathan and Grace 2018). However, in this passive targeting mode, liposomes frequently encounter obstacles in clinical environments due to the off‐target effect and RES, which swiftly eliminate the liposomal system from blood circulation (Daraee et al. 2016).

**FIGURE 7:**
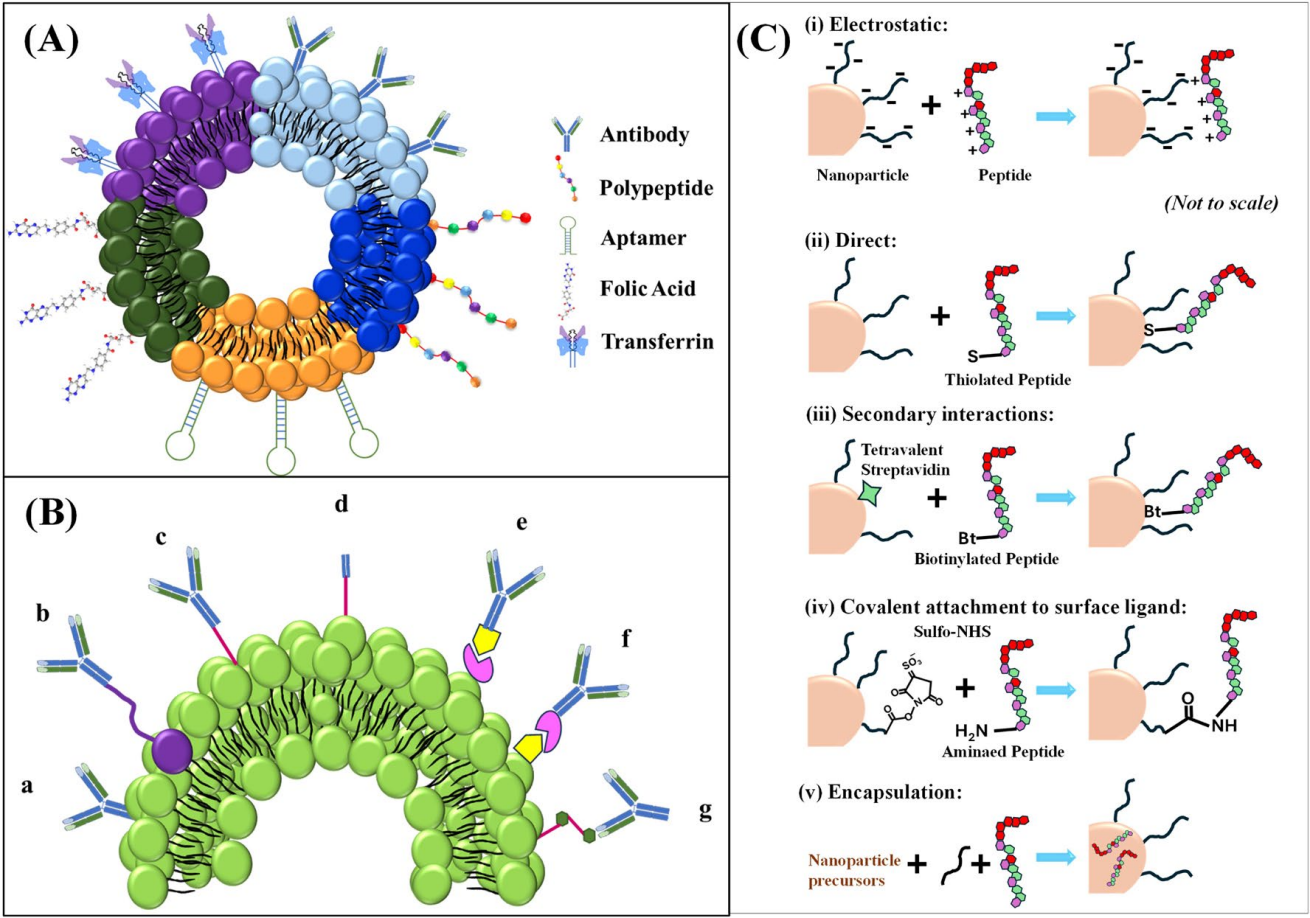
(A) Surface‐coated biomimetic liposome nanoparticle. The surface of the liposome is commonly functionalized with antibodies, polypeptide, aptamer, folic acid, and transferrin to enhance its targeting property; (B) Antibody‐coated immune liposomes. Various ab and ab‐fragments conjugated with surface phospholipid of liposomes by employing different techniques: (a) direct adsorption; (b) binding of whole ab by utilizing PEG spacers; (c) covalent conjugation of whole ab; (d) covalent conjugation of ab fragment (Fab’); (e, f) whole ab conjugation through avidin–biotin with either the biotin or avidin bound to the liposome surface; (g) conjugation through hapten; (C) Peptide tagging on the surface. (i) electrostatic conjugation. (ii) direct conjugation. (iii) secondary interaction. (iv) covalent attachment to surface ligand. (v) encapsulation or fusion.

The second and most efficient mode of targeting liposomes is the active targeting mode. Considering the different constraints of the passive targeting mode of liposomes, the development of unique approaches resulted in the discovery of active targeted liposomes (Byrne et al. 2008). Active targeting denotes the process of altering the outer surface of liposomes by conjugating the targeting moieties on the phospholipid bilayer, including aptamers, small molecules, antibody fragments, or whole antibodies, peptides, and other compounds that can specifically target and bind to the receptors found on the desired cells (Alghamdi et al. 2022; Khan et al. 2020). This approach improves the precision and effectiveness of cargo material distribution. These liposomes are specially designed to lessen the off‐target effects. Surface modification of the liposome is one of the best methods to enhance its stability and target site reachability. Surface coating of liposomes plays a vital role in improving their performance and ability to interact with biological systems. Various coating strategies have been explored to improve the efficient pharmacokinetic and pharmacodynamic characteristics of liposomes (de Leo et al. 2021). Hereby, we are discussing the surface modification strategies to develop biomimetic liposomal NPs with specific coating materials such as antibodies, polypeptides, aptamers, folic acid, and transferrin (Figure 7A).

### 3.1 Antibody Coated Liposomes

The liposomes conjugated with antibodies on the surface are also called immunoliposomes, which are engineered biomimetic NPs to actively target specific sites and deliver the drugs efficiently (Hama et al. 2021; Li et al. 2021). Antibodies play a vital function in targeted drug delivery systems by enhancing their selectivity and specificity for cancer treatment and diagnostic applications. Engineering drug delivery systems with antibodies as bioactive agents facilitates the precise delivery of drugs to target sites (Kaneko 2023). The conjugation of antibodies on the surface of the liposome is one of the promising approaches for targeted drug delivery. Several studies have explored various methods to effectively accommodate antibodies onto the surface of the liposomes (Figure 7B; Di et al. 2020; Hama et al. 2021; Safari et al. 2024). The level of antibody coating on the liposomal surface is essential for their functionality. Researchers have demonstrated that a larger density of antibodies covering a surface can result in enhanced activation of the complement system, increased concentration of blood plasma, higher numbers of white blood cells, and improved removal of substances by the liver (Tan 2022). In addition, the valency of antibodies on liposomal surfaces disturbs their binding abilities, and the density of surface ligands is a crucial factor in determining their binding capabilities (Zhou et al. 2023). Furthermore, the process of altering antibodies on the liposomal surface can be accomplished quickly by utilizing liposomes that display high‐affinity protein. This permits the targeted drug administration based on the modified antibodies. Optimizing the concentration and valency of antibody coating on the liposomal surface is crucial for maximizing the effectiveness of targeting ability, reducing the side effects, and improving cargo delivery (Hardy and Dimmock 2003; Ho et al. 2024; Mohammad Faizal et al. 2023).

Antibodies are functional molecules composed of two sets of light and heavy chains linked by intrachain disulfide bonds. Due to their immunologic properties, these are revolutionized in the diagnostics and therapeutics fields (Daly 2022). Antibodies are categorized into different classes based on their structure and function. The major classes of immunoglobulins are IgA, IgD, IgE, IgG, and IgM (Parija 2023). IgG is the prime choice for conjugation with liposomes due to its structural property, and IgM is used in some formulations. The fundamental architecture of an IgG antibody molecule is composed of four polypeptide chains with two light and two heavy chains linked together by disulfide bonds.

Epidermal growth factor receptor (EGFR) is a cell surface receptor, highly expressed in several cell types, especially in cancer cells (Byeon et al. 2019; Ciardiello and Tortora 2003; Normanno et al. 2006). Several research studies demonstrate that EGFR overexpression correlates with cancer cell differentiation and migration. Cetuximab, a chimeric monoclonal antibody, selectively binds to EGFR, inhibiting its activation and subsequent signaling pathways, hence affecting cancer development and progression. Cetuximab is the predominant anti‐EGFR antibody employed for the alteration of nanocarrier surfaces. McDaid et al. targeted EGFR overexpression and Cetuximab (CTX) resistant cancer cells with cetuximab when utilized with camptothecin‐loaded polymer NPs. This study found that the CTX‐coupled nano drug delivery system improved NPs' cell surface targeting via interacting with EGFR. Further research indicates that the CTX‐modified nano‐drug carrier system enhances tumor suppression and targeting (McDaid et al. 2019).

### 3.2 Polypeptide‐Coated Biomimetic Liposome Nanoparticles

Peptides are tumor‐specific ligands made up of less than 50 amino acids. They possess a small size, excellent affinity, good stability, ease of modification, and minimal immunogenicity. They have become more common in the field of tumor diagnosis and treatment. Peptides have garnered considerable attention in biological applications, including drug delivery, cancer therapy, and vaccine creation, owing to their specificity, low toxicity, and biocompatibility. Peptides have numerous obstacles, such as inadequate stability, vulnerability to proteolytic degradation, and restricted bioavailability, which impede their therapeutic application (Nhàn et al. 2023; Samec et al. 2022; Sonju et al. 2021).

*Electrostatic tagging*: The interaction between cationic peptides and negatively charged liposomes is a simple and traditional method for forming stable peptide‐liposome complexes. This technique utilizes electrostatic interactions, which are straightforward and efficient for coating applications. The stability of these compounds can be affected by environmental conditions, including ionic strength and pH. Among various peptides, RGD (arginine–glycine–aspartate) is pivotal in targeting integrin receptors, especially integrin αvβ3, which are overexpressed on tumor endothelial cells and are integral to tumor angiogenesis (Chen et al. 2009). Cationic liposomes, owing to their positive charge, can electrostatically bind with negatively charged RGD peptides, resulting in the formation of a stable peptide–liposome complex (Figure 7C; Sapra and Allen 2003).

*Covalent attachment*: Covalent conjugation of peptides to the liposome surface enhances stability and prevents premature release. Common methods include attaching peptides through functional groups like amines, carboxyl, or thiol groups to reactive moieties on the liposome surface. This method provides a stronger and more stable interaction, ensuring better control over peptide release and reducing the risk of dissociation in the bloodstream (Figure 7C; Gyongyossy‐Issa et al. 1998; Liu et al. 2021; Taneichi et al. 2006).

*Lipid anchoring*: Lipid‐modified peptides can be anchored into the liposome bilayer through hydrophobic interactions. Peptides can be conjugated with lipid moieties, such as fatty acids or cholesterol, allowing them to insert into the liposomal membrane. This method enhances the stability of peptide–liposome formulations, facilitates membrane fusion, and improves cellular uptake (Figure 7C; Dissanayake et al. 2022; Nsairat et al. 2022).

*PEGylation*: Polyethylene glycol (PEG) can be conjugated to peptides and used to coat liposomes, offering steric stabilization and reducing immune recognition. PEGylation prolongs circulation time by reducing opsonization and clearance by the reticuloendothelial system (RES; Askarizadeh et al. 2024; Xia et al. 2023). It also provides a platform for attaching targeting ligands or peptides, which can improve the specificity of liposomal delivery to certain tissues or cells, such as tumors (Figure 7C; Mehrizi et al. 2024; Suk et al. 2016).

*Fusion peptides*: Fusion peptides can be designed to facilitate the interaction of liposomes with target cells or tissues. These peptides can incorporate sequences that promote cell penetration, receptor‐targeting, or membrane fusion. Such fusion peptides are often embedded into the liposomal bilayer, improving the delivery of encapsulated drugs or cargo through enhanced cellular internalization or endosomal escape (Figure 7C; Iversen et al. 2024; Zeng et al. 2023).

### 3.3 Aptamer‐Coated Biomimetic Liposome Nanoparticles

Aptamers are short single‐stranded DNA or RNA oligonucleotides, typically comprising 25 to 90 nucleotide bases, that attach to specific targets such as proteins and cells via distinct three‐dimensional conformations (Kar 2024; Zhou, Li, and Wu 2024). Aptamers have emerged as exceptional and rapidly developing tools for successfully targeting cancer biomarkers and are utilized as effective ligands for drug delivery and anti‐cancer therapy. These ligands tend to have the ability to bind with the nanomolar to the picomolar range of target molecules with precise binding affinities (Safarkhani et al. 2024).

Aptamers are generated using the systematic evolution of ligands by exponential enrichment (SELEX) technology, which precisely binds a wide array of target materials, including cells, viruses, proteins, and small molecules (Brown et al. 2024). Nucleic acid aptamers have surpassed antibodies for the targeted delivery of anticancer medicines. Aptamers are useful ligands due to their resistance to organic solvents, temperature fluctuations, and pH variations, as well as their capability for mass manufacturing by chemical synthesis. Aptamers exhibit resistance to denaturation–renaturation cycles and possess lower immunogenicity compared to antibodies (Erkmen et al. 2024; Ni et al. 2021).

Aptamer‐conjugated liposomes represent the most effective drug delivery technique. The US FDA has sanctioned several liposome‐based therapeutics across various clinics for disease treatment (Jiang et al. 2024; Wong et al. 2024). Aptamers are chemically modified with diverse functional groups at both termini to improve site‐specific conjugation. Nucleic acid aptamers demonstrate rapid infiltration into the target cells and enhanced serum retention owing to superior stability (Gao et al. 2024; Malone et al. 1989; Miller et al. 2017; Zhou, Li, and Wu 2024). The proliferation and spread of tumors are facilitated by some immunosuppressive cells, including tumor‐associated macrophages, myeloid‐derived suppressor cells, and tumor‐resident regulatory T cells (Ghebremedhin et al. 2024; Park et al. 2024). Targeting these immunosuppressive cells can enhance the effectiveness of cancer treatment.

Various research works demonstrated that the RNA aptamer (Interleukin‐4 receptor subunit alpha (IL‐4Ra)) inhibits the human IL‐4 receptor (Roth et al. 2012; Sharif‐Askari et al. 2024) and concurrently suppresses myeloid‐derived suppressor cells (Liu et al. 2017). CpG (Cytosine‐phosphate‐Guanine) oligodeoxynucleotide‐10 demonstrated remarkable anti‐tumor activity as an aptamer, although the IL‐4Ra‐liganded liposome precisely targets the IL‐4Ra receptor present on CT26 carcinoma cells, which shows a significant expression of the IL‐4Ra receptor (Liu et al. 2017; Loira‐Pastoriza et al. 2021). The effective uptake of CpG by tumor cells markedly hinders in vivo CT26 tumor growth, and the delivery of this cancer‐targeting aptamer through liposomes may provide a formidable approach to surmount immunosuppression and augment immunotherapy (Kim, Lee, and Jon 2024; Wang, Chen, et al. 2023).

### 3.4 Vitamins‐Coated Biomimetic Liposome Nanoparticles

The utilization of vitamins as a surface coating ligand for liposomes has enhanced their utility in customized cargo delivery (Misra and Pathak 2022; Patel et al. 2024). Different types of cancer cells exhibit a higher expression of vitamin receptors compared to normal cells, thus necessitating a comprehension of these receptors for the effective docking of vitamin‐liganded liposomes. Malignant phenotypes frequently demonstrate elevated expression of several vitamin receptors (Dinakar et al. 2023; Kułdo et al. 2005; Soe et al. 2018). The predominant liposome ligands for malignant cell receptors are folate; however, tocopherol, pyridoxal phosphate, and pyridoxine have also been utilized (Dinakar et al. 2023; Khan et al. 2020; Kumar et al. 2022). Vitamin E can be used as a liposomal ligand to target diseased cells in the form of d‐alpha tocopheryl polyethylene glycol succinate (TPGS). This amphiphilic structure has PEG as the hydrophilic element and tocopherol succinate as the lipophilic moiety. TPGS's hydrophilic–lipophilic balance renders it an efficient solubilizer, emulsifier, and bioavailability enhancer for hydrophobic pharmaceuticals (Duhem et al. 2014; Jasim et al. 2021; Yang et al. 2018). Pérez‐Herrero and Fernández‐Medarde (2015) assert that TPGS enhances drug absorption, cytotoxicity, and reduces multidrug resistance (Pérez‐Herrero and Fernández‐Medarde 2015). Vitamin E is employed to safeguard liposomes from free radical damage and to mitigate oxidative stress during storage (Kilicarslan You et al. 2024; Suntres 2011).

During carcinogenesis, overexpression of folate receptors occurs in their plasma membranes, which is especially a special affinity receptor for folic acid (Bertel et al. 2024; Gonzalez et al. 2024; Paulos et al. 2004). One of the commonly approached methods for conjugating folic acid to the liposomes involves the preparation of folate‐linked peptides, cholesterol, or phospholipids before developing tumor‐specific liposomes (Kumar et al. 2019; Nogueira et al. 2015; Shmendel et al. 2023). Normal tissues typically exhibit low or lack folate receptor expression on cells (Varaganti et al. 2023). The selective expression of folic acid receptors in cancer cells has been utilized as a signal marker for liposomes to actively target and deliver treatment and diagnostic cargo into the cancer cells (Nehal et al. 2024; Wen et al. 2024). Numerous chemotherapeutics and diagnostic imaging agents target tumor cells through a folate receptor binding mechanism (Cheung et al. 2016; Fernández et al. 2018; Ledermann et al. 2015; Xu et al. 2017). In an earlier stage of folate‐mediated liposome preparation, phospholipids were directly conjugated with folic acid. Later research works demonstrated that tumor cells' folate receptors did not engage with folate‐conjugated liposomes when folic acid was directly bound to phospholipids (Drummond et al. 2000; Kumar et al. 2019). To focus on this constraint, linkers like polyethylene glycol (PEG) and hydrazine were used to achieve the direct binding of folic acid to protein, cholesterol, and other spacers (Aucoin et al. 2024; D'Souza and Shegokar 2016; Sampogna‐Mireles et al. 2017). Tang and coworkers demonstrated that using PEG in folate‐conjugated liposomes boosted therapeutic effectiveness by increasing solubility, half‐life, and drug reserve at the tumor site (Tang et al. 2023). Folic acid conjugation with PEG for liposome development improved liposome retention in the tumor sites and paved the path for endocytosis via folate receptors (Lim et al. 2023).

### 3.5 Effect of Liposomal Drug Delivery Systems

**FIGURE 8:**
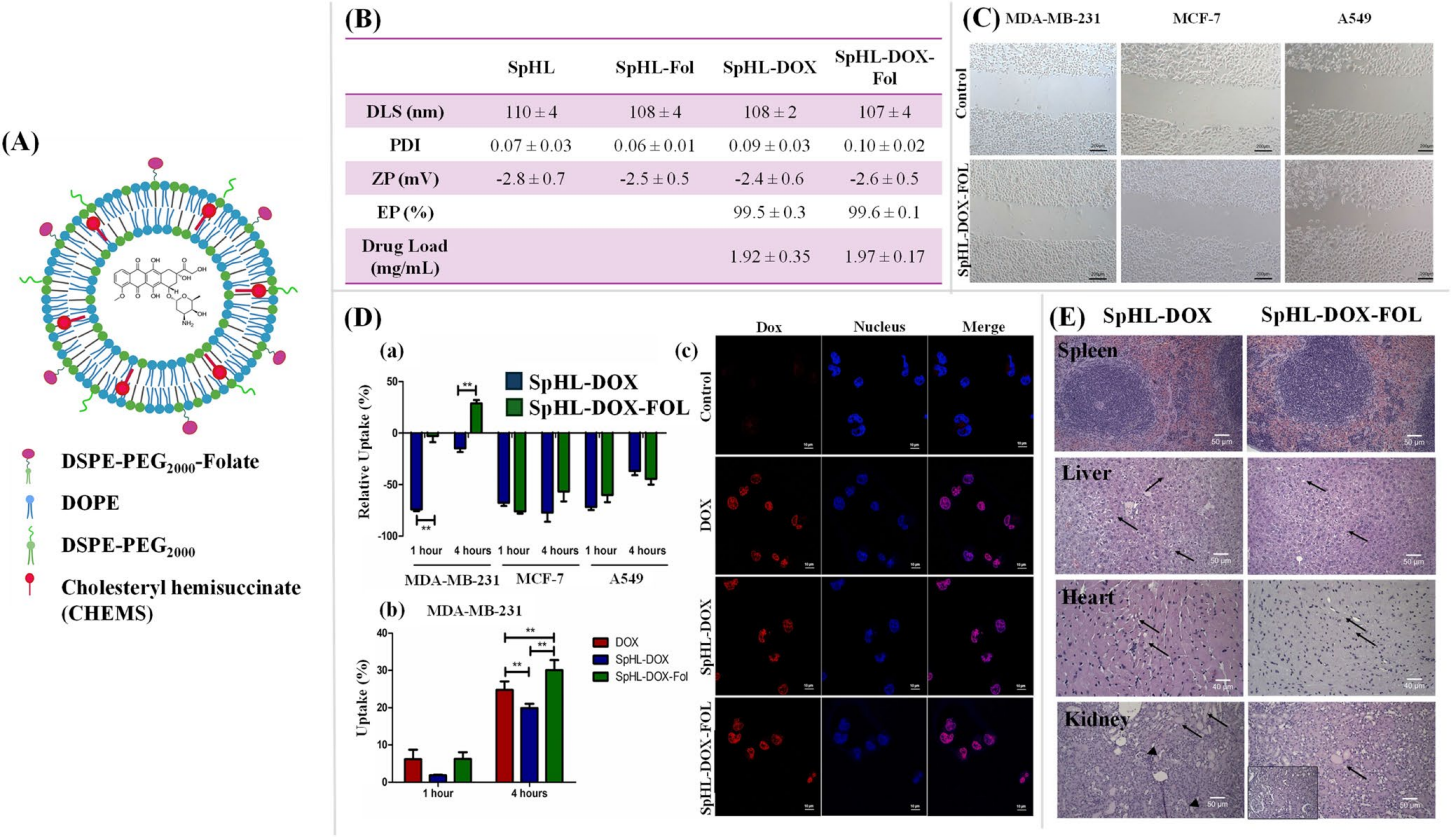
Acute toxicity and in vitro antitumor activity of Dox‐loaded folate‐coated liposomes (SpHL‐DOX‐FOL). (A) Model illustration of the anatomy of the nanoparticle. (B) Characterization of free liposomes and drug‐loaded liposomes. (C) In vitro cell migration assessment. (D) In vitro cellular uptake of DOX SpHL‐DOX‐FOL. SpHL‐DOX‐FOL showed enhanced internalization as compared to that of other treatment groups. (E) Histopathological evaluation of various tissues treated with SpHL‐DOX and SpHL‐DOX‐FOL (Reprinted with permission from de Oliveira Silva et al. (2023). Copyright 2023 Elsevier Ltd).

Based on various synthesis and surface coating strategies, numerous liposomal drug delivery systems were developed for cancer therapeutics and diagnostics applications. The liposome, which is coated with folic acid to target folate receptors, is one of the effective drug delivery systems to target cancer cells that are highly expressed with folate receptors. Oliveira and the team have investigated the efficacy and safety of a folate‐coated doxorubicin‐loaded pH‐sensitive liposome (SpHL‐DOX‐Fol) to improve doxorubicin (DOX) administration to folate receptor‐positive (FR+) cancer cells. The researchers analyzed the impact of folate functionalization on DOX delivery to breast cancer (MDA‐MB‐231, MCF‐7) and lung cancer cells (A549). The study shows that SpHL‐DOX‐Fol has a much higher cellular uptake and cytotoxicity against FR+ MDA‐MB‐231 breast cancer cells than nontargeted SpHL‐DOX and free DOX. This increased efficacy is related to the folate‐mediated endocytosis mechanism, which results in an IC_50_ of 387 nM for SpHL‐DOX‐Fol, significantly lower than that of SpHL‐DOX (450 nM) and free DOX (518 nM). In vivo acute toxicity tests in BALB/c mice revealed that SpHL‐DOX‐Fol efficiently reduced systemic and cardiotoxicity compared to free DOX. Animals treated with SpHL‐DOX‐Fol had considerably decreased creatine kinase‐MB (CK‐MB) levels, less hepatic and renal toxicity, and few histopathological changes in cardiac and renal tissues. Importantly, SpHL‐DOX‐Fol had improved hematological profiles, with no significant leukopenia or thrombocytosis, unlike free DOX‐treated groups. These findings underline SpHL‐DOX‐Fol's improved therapeutic efficacy and safety profile, indicating its promise as a promising nanocarrier for targeted cancer therapy, particularly for FR+ breast cancers. This study provides a solid foundation for future translational research and clinical uses of folate‐functionalized liposomal systems (Figure 8; de Oliveira Silva et al. 2023).

**FIGURE 9:**
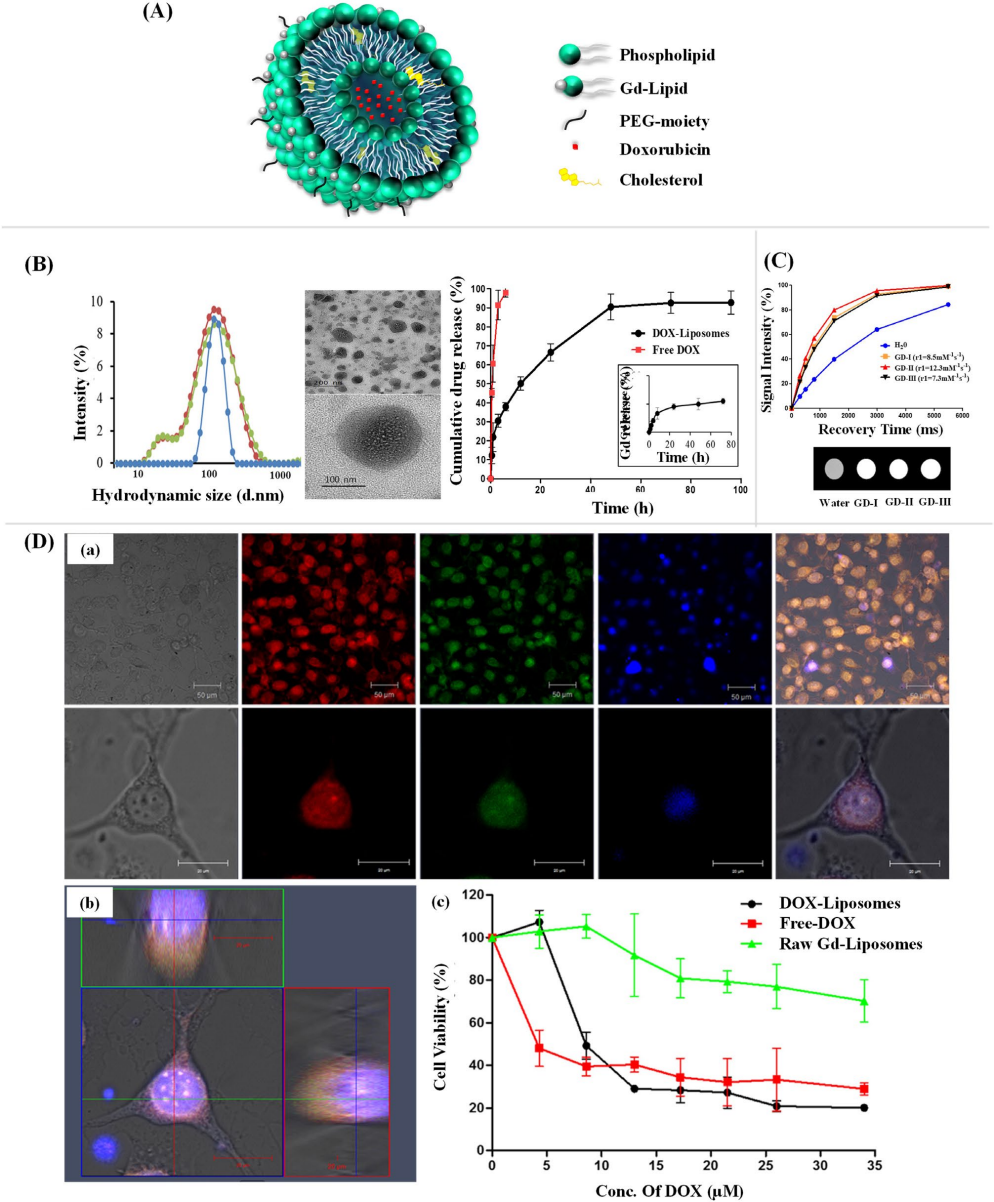
Gadolinium‐infused theranostic liposomes. (A) Model illustration of nanoparticles. (B) Characterization of DOX‐Gd‐Liposomes using DLS, TEM, and in vitro drug release assays. The DOX‐loaded Gd liposomes showcase the efficient physiological features. (C) Magnetic properties of Gd‐liposomes. Contrast phantoms of infused theranostic liposomes acquired at 14.1 T. (D) In vitro cytotoxicity and cellular uptake of DOX‐Gd‐Liposomes. (Reprinted with permission from Pitchaimani et al. (2016). Copyright 2016 The Royal Society of Chemistry).

PEG coating is a pivotal strategy in the development of liposome‐based drug delivery systems for enhanced drug delivery and safety profiles. The PEGylated and gadolinium‐infused theranostic liposome was developed by Pitchaimani and coworkers to improve diagnostic and therapeutic capabilities against various cancer types. The team ingeniously incorporates gadolinium ions into the hydrophilic heads of phospholipids, improving the liposomes' structural stability and magnetic features. The liposomes exhibited a homogenous spherical vesicle with a hydrodynamic size of 150 ± 10 nm and also showcased enhanced loading of doxorubicin and sustained drug release (Figure 9A,B). In comparison with the conventional contrast agent Magnevist, the Gd‐infused liposomes displayed three times higher T1 relaxivity (12.3 mM^−1^ s^−1^ at 14.1 T; Figure 9C). These meticulously designed liposomes not only maintain stable and uniform size distribution but also exhibit significantly higher relaxivity than traditional gadolinium‐based treatments. This increase potentially improves the quality of MRI images (Figure 9C). The cellular uptake study undertaken with B16F10 melanoma cells demonstrated consistent intracellular distribution, while in vitro cytotoxicity assays revealed comparable therapeutic effects to free DOX (Figure 9D). These results strongly suggested that the developed PEGylated liposome has considerable potential as a theranostic nanosystem for clinical applications, especially in targeted cancer therapy and diagnostics. The study is well‐structured, presenting a clear methodology and substantial evidence supporting the therapeutic and diagnostic superiority of gadolinium‐infused liposomes over conventional approaches (Pitchaimani et al. 2016).

**FIGURE 10:**
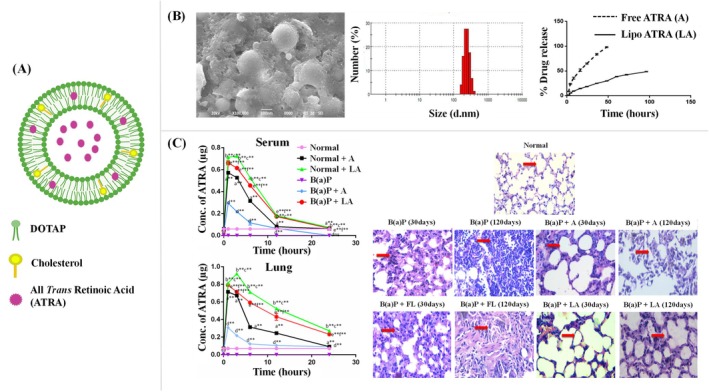
Cationic liposome system loaded with All *trans* Retinoic Acid (ATRA) for lung cancer treatment. (A) Model illustration of nanoparticles. (B) TEM, DLS, and drug release profile of Lipo‐ATRA. Characterization studies show a gradual drug release profile. (C) In vivo pharmacokinetics and anti‐cancer studies of Lipo‐ATRA reveal the efficient therapeutic efficacy. (Reprinted with permission from Berlin Grace and Viswanathan (2017). Copyright 2017 Elsevier Ltd).

Berlin Grace and coworkers successfully developed cationic liposome NPs loaded with all‐*trans*‐retinoic acid (ATRA) and studied their pharmacokinetics and therapeutic function against chemical carcinogen‐induced animal models (Berlin Grace and Viswanathan 2017). The study demonstrates the successful formulation of a cationic liposome‐based nano‐delivery system, addressing key limitations of ATRA, such as poor bioavailability and rapid degradation. Pharmacokinetic analysis reveals significant improvements in drug stability, systemic circulation time, and targeted delivery to tumor sites. The therapeutic efficiency, evaluated in vivo using a lung cancer mouse model, highlights a marked reduction in tumor growth and enhanced survival rates compared to conventional ATRA treatment. Additionally, the paper provides compelling evidence of reduced systemic toxicity, underscoring the formulation's safety profile. This innovative work not only advances the field of nanomedicine but also witnesses the potential of liposomal delivery systems for improving cancer therapy outcomes (Figure 10; Berlin Grace and Viswanathan 2017).

## 4 Extracellular Vesicle and Liposome Hybrid System

EVs are essential for intercellular communication, facilitating the transport of bioactive substances, including proteins, nucleic acids, and lipids, between cells. EVs have garnered considerable attention as prospective carriers for therapeutic agents, especially in drug administration, cancer treatment, and regenerative medicine, owing to their inherent biocompatibility, capacity to navigate biological barriers, and natural targeting abilities.

EVs from cells are an emerging alternative to nanoparticle drug delivery systems due to their biological origin and targeting capabilities (Debbarma et al. 2024; Sanyal and Banerjee 2024; Ubanako et al. 2024). They serve crucial functions in cellular communication, genetic material transfer, and immune response regulation (Aloi et al. 2024; Essola et al. 2023; Sunkara et al. 2025). The study on EVs biogenesis, isolation, and characterization has been extensively undertaken by various researchers (de Sousa et al. 2023; Miron and Zhang 2024; Salmond and Williams 2021; Sani et al. 2024; Sonbhadra et al. 2023). EVs have more complicated lipid components than liposomes, affecting their physical characteristics and interactions with recipient cells (Skotland et al. 2020). Several proteins have been found to be incorporated or linked to the EV membrane. The presence of molecules like integrins, tetraspanins, and proteoglycans may contribute to their biocompatibility, stability, targeting specificity, and ability to cross biological barriers (Alvarez‐Erviti et al. 2011; Millard et al. 2018; Murphy et al. 2019; Schindler et al. 2019). However, the intricacy of EV surfaces limits drug loading. Two main ways for loading therapeutic cargo into EVs are endogenous (passive) and exogenous (e.g., electroporation).

**FIGURE 11:**
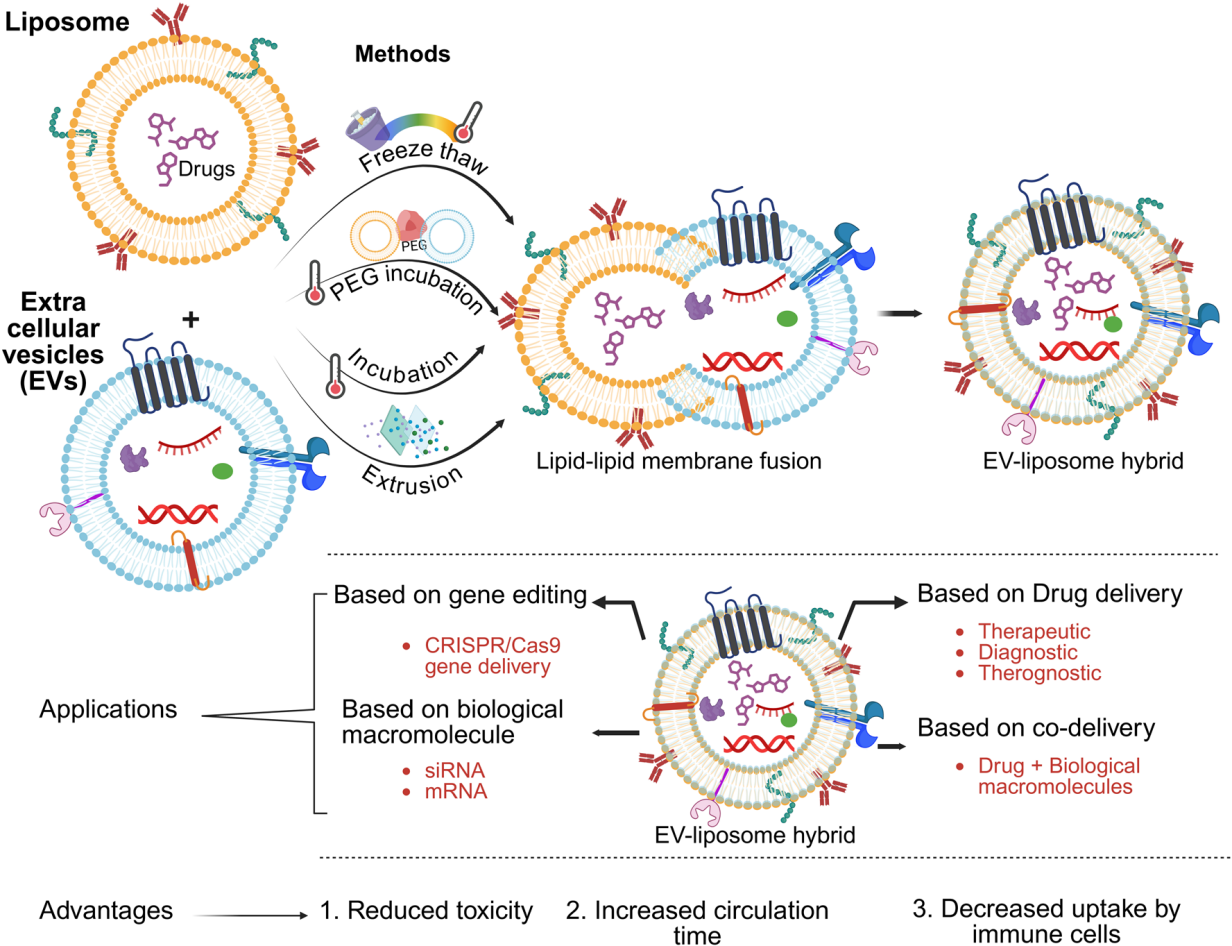
Synthetic strategies and overview of hybridized biomimetic EVs. EVs are commonly hybridized with synthetic nanoparticles using freeze–thaw, PEG incubation, Incubation, and extrusion strategies.

Liposomes are synthetic spherical vesicles made of phospholipid bilayers. They have been utilized in drug delivery for an extended period because of their capacity to encapsulate both hydrophilic and hydrophobic pharmaceuticals, enhancing their stability, biodistribution, and bioavailability (Dymek and Sikora 2022; Mehta, Bui, et al. 2023; van der Koog et al. 2022). Liposomes are adaptable and can be modified with ligands or peptides to improve targeting and therapeutic effectiveness, as discussed above. Membrane fusion‐based hybrid exosomes (MFHE) are a new nanoparticle for drug administration that combines the benefits of liposomes and exosomes through various membrane fusion mechanisms (Lu and Huang 2020). MFHEs possess strong drug loading, stability, and surface modification capabilities, as well as high biocompatibility and low exosome immunogenicity. This sheds light on nanoparticle medicine delivery (Figure 11).

### 4.1 Synthetic Strategies

Various synthetic strategies were developed to engineer EVs and liposome hybrid systems as discussed in the table below (Table 4).

**TABLE 4:** Technical strategies, advantages, and disadvantages of EV and liposome hybridization techniques.

Methods	Technical procedure	Advantages	Disadvantages	References
Extrusion	Co‐extrusion of the EVs with liposomes through a polycarbonate membrane of defined pore size (disrupts the lipid layers, transiently utilizing the physical forces)	Rapid technique Relatively higher efficiency	Potential damages arise to the EVs membrane Relatively complicated procedure	(Evers et al. 2022; Hu et al. 2021; Rayamajhi et al. 2019; Sulthana et al. 2024)
Freeze–thaw	Hybridization undertaken by freezing the mixture of EVs and liposomes repeatedly (transient disruption of the lipid layers through the formation of ice crystals)	Rapid and easy procedure Relatively high efficiency	Loss of drug Disruption of EVs membrane Potential leakage and loss of the components	(Cheng et al. 2021; Kannavou et al. 2021; Lv et al. 2020; Sato et al. 2016; Singh et al. 2021)
Incubation	Incubated basis hybridization was undertaken by keeping the mixture of EVs and liposomes at 37°C (due to the lipid structure of these two NPs)	Straightforward Maintenance of EVs and liposome membranes	Lesser fusion efficiency Time taking process Restrictions by physicochemical properties of vesicles	(Lin et al. 2018)
PEG Incubation	With the help of PEG incubation, the fusion between EVs and liposomes has been undertaken (facilitates tight contact of lipid bilayers and triggers protein‐free membrane fusion)	Straightforward Maintenance of EVs and liposome membranes Significant prolongation of the MFHEs blood circulation time	Time‐taking method Negative effect on cellular uptake	(Kannavou et al. 2021; Ma et al. 2022; Piffoux et al. 2018)

#### 4.1.1 Extrusion Methods

The membrane extrusion method involves the concurrent ejection of exosomes and liposomes via membrane pores of adjustable dimensions under applied physical pressure to create mixed vesicles. In comparison to incubation and freeze–thaw techniques, membrane extrusion offers the benefit of achieving a more uniform particle size in hybrid vesicles. Sun et al. created hybrid nanovesicles utilizing clodronate‐loaded (CLD) liposomes and exosomes produced from fibroblasts for the therapeutic intervention of pulmonary fibrosis. The researchers combined L‐929 (Murine fibroblast cell line) fibroblast‐derived exosomes with a suspension of synthetic liposomes at a 1:5 protein equivalent ratio, vortexed and sonicated the mixture, and subsequently extruded it through 400 and 200 nm polycarbonate ester membranes 10 times. This led to the effective creation of exosome‐hybridized liposomes (Sun et al. 2021).

Other research teams synthesized hybrid vesicles using analogous techniques. Liposome and exosome solutions were combined in different volumetric proportions. Thereafter, the mixtures were typically vortexed and sonicated for 2–3 min using a sonicator set at 20%–33% of its maximum amplitude to achieve complete solvation of the solution. The mixes were extruded via pore diameters of 400, 200, or 100 nm (Evers et al. 2022; Hu et al. 2021; Jhan et al. 2020; Li, He, et al. 2022; Rayamajhi et al. 2019). The pore size of the polycarbonate membrane and the frequency of membrane extrusion influence the characteristics of MFHEs. While membrane extrusion techniques exhibit elevated fusion efficiency, the shear stress produced during the extrusion process may compromise the structural integrity of natural exosomes.

#### 4.1.2 Freeze–Thaw Methods

Freeze–thaw procedures are routinely used to load drugs onto liposomes. Creating ice crystals can rupture the plasma membrane and allow water‐soluble compounds to enter liposomes (Roque et al. 2023). This procedure can also be used to create hybrid EVs. Many research teams have achieved good fusion efficiency while using varied numbers of freeze–thaw cycles. Sato et al. combined Raw 264.7 cell‐derived exosomes with dual fluorescently labeled liposomes (1:1 by volume). After freezing in liquid nitrogen, the mixture was thawed at ambient temperature for 15 min. The hybrid exosomes produced after repeated freeze–thaw cycles have a greater cellular absorption rate than liposomes (Sato et al. 2016).

Cheng et al. created hybrid exosomes by combining genetically altered exosomes with heat‐sensitive liposomes for cancer treatment using photothermal therapy and immunotherapy. Researchers created exosome–liposome hybrid NPs by mixing heat‐sensitive liposomes and genetically altered exosomes at a 1:1 ratio and freezing–thawing them three times. The fusion efficiency of this synthetic approach reached 97.4% (Cheng et al. 2021).

#### 4.1.3 Natural Incubation

Membrane fusion is an autonomous process that employs the physicochemical properties of vesicles to facilitate fusion. Hybrid exosomes are generated via electrostatic or hydrophobic interactions, maintaining the integrity of the lipid bilayer and preventing the leakage of vesicular contents. Lin et al. synthesized hybrid exosomes by incubating HEK293FT cell‐derived exosomes with CRISPR/Cas9‐expressing liposomes at 37°C for 12 h, presenting a novel approach for the secure and efficient delivery of the CRISPR–Cas9 system (Lin et al. 2018). This technique inflicts minimal harm to vesicles and pharmaceuticals. However, the fusion efficiency is comparatively poor.

#### 4.1.4 Polyethylene Glycol Incubation

Polyethylene glycol (PEG) alters cell membranes and is extensively utilized to facilitate cell‐to‐cell fusion by promoting the proximity of lipid bilayer membranes and initiating the displacement and restructuring of lipid molecules (Yoshihara et al. 2020). Piffoux et al. revealed that PEG might facilitate the fusion of exosomes and liposomes derived from several cellular sources. The fusion effectiveness of liposomes and exosomes was assessed using various ratios, sizes, and concentrations of PEG molecules (Piffoux et al. 2018). Their findings demonstrate a more efficient fusion of 30% (v/w) PEG 8000. Due to its facile preparation and stable activity, PEG can facilitate the effective fusion of exosomes and liposomes while also prolonging their circulation time in the bloodstream. Nonetheless, the presence of PEG on the surface of hybrid exosomes may be inadequate to confer the stealth characteristics necessary to evade swift reception by the RES, therefore diminishing the cellular uptake of the hybrid exosomes (Kannavou et al. 2021; Lee et al. 2021; Patras et al. 2022).

### 4.2 Effect of Hybridized Biomimetic Nanodrug Delivery Systems

**FIGURE 12:**
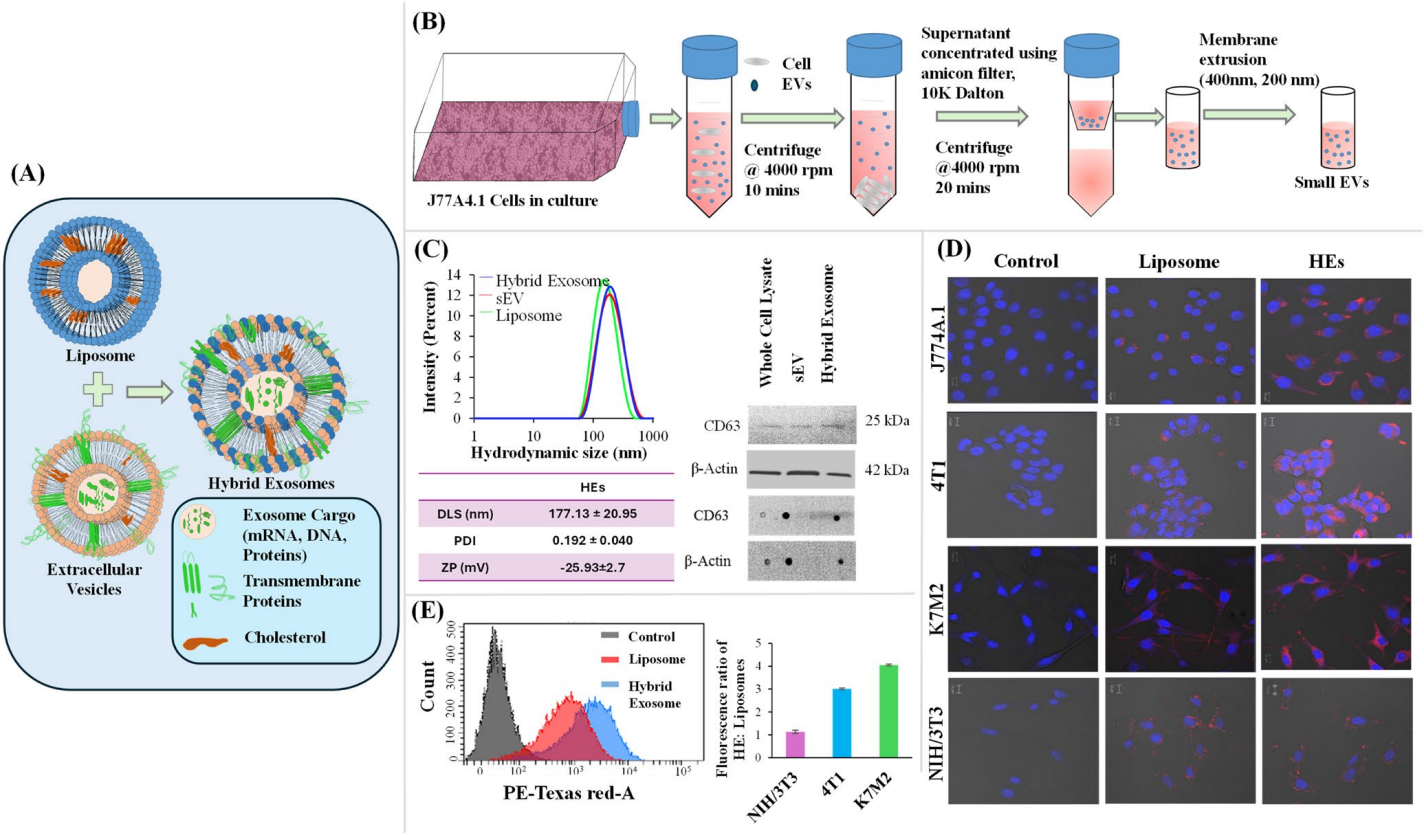
Hybridization of macrophage‐derived EVs for targeted drug delivery. (A) Schematic of hybrid exosomes (HEs). (B) Hybridization of EVs with liposomes. (C) Physico‐chemical properties assessment for HEs. The DLS studies showed the optimum physical properties, and dot blot studies showed the presence of bio‐markers in the vesicles which further revealed the efficient hybridization. (D, E) In vitro cellular uptake studies showed the active internalization of HEs over the liposomes. (Reprinted with permission from Rayamajhi et al. (2019). Copyright 2019 Elsevier Ltd).

Hybrid EVs represent a novel and innovative frontier in the area of nano‐engineered drug delivery systems, utilizing the intrinsic biological features of natural EVs with the tailored properties of synthetic materials to enhance therapeutic efficacy and targeting specificity. With this idea, there are numerous research works carried out to engineer innovative hybridized vesicles for effective therapeutic and diagnostic applications. The study by Rayamajhi and colleagues explores the synthetic strategy of hybrid vesicles that combine the benefits of macrophage‐derived EVs and synthetic liposomes, with the objective of leveraging the biological targeting potential of EVs while improving drug delivery efficiency through the adaptable nature of liposomes (Figure 12A). The research group successfully hybridized the EVs with synthetic liposomes to generate the hybrid exosomes (HEs) that retained the size characteristics beneficial towards biological applications (those less than 200 nm hydrodynamic diameter). The critical aspect of their methodology was confirming that these HEs retained the surface proteins required for targeting tumor cells, a characteristic inherited from their parental macrophage origin. This research work thoroughly characterized these hybrid vesicles employing various techniques such as dynamic light scattering and transmission electron microscopy, demonstrating their anticipated size distribution and morphological integrity (Figure 12B,C). The researchers emphasized the stability of these HEs, which is a notable advancement over conventional EVs, as it could lead to more predictable and controlled therapeutic outcomes. This study's one of the most compelling aspects is its assessment of the targeted drug delivery potential utilizing the in vitro models. To achieve this, the vesicles were loaded with doxorubicin, a commonly recognized chemotherapy drug. The results showed that these HEs could target the interested cancer cells and deliver their cargo directly into the destination cells while limiting exposure to normal cells (Figure 12D,E). The selective toxicity was significantly higher in cancer cells than in noncancerous cells, showing the vesicles' potential to effectively target and kill tumor cells. The drug release experiments emphasized the HEs' ability for controlled release, exhibiting a pH‐sensitive release beneficial in the acidic tumor microenvironment commonly present in cancer sites. This feature may diminish chemotherapy side effects by confining drug release to the target site, thus shielding healthy tissues from exposure to hazardous chemotherapeutic agents. In conclusion, this study notably contributed to the field of drug delivery, especially in the development of innovative vesicle‐based systems for cancer therapy and diagnostics. By combining the natural targeting features of EVs with the robustness and flexibility of liposomes, the study not only addresses some of the key challenges in the field of EVs but also opens new avenues for the targeted treatment of cancer. The capability of these hybrid exosomes to improve the therapeutic index of chemotherapeutic drugs may have substantial significance for future clinical applications, representing progress in the search for more effective and less harmful cancer treatments (Rayamajhi et al. 2019).

**FIGURE 13:**
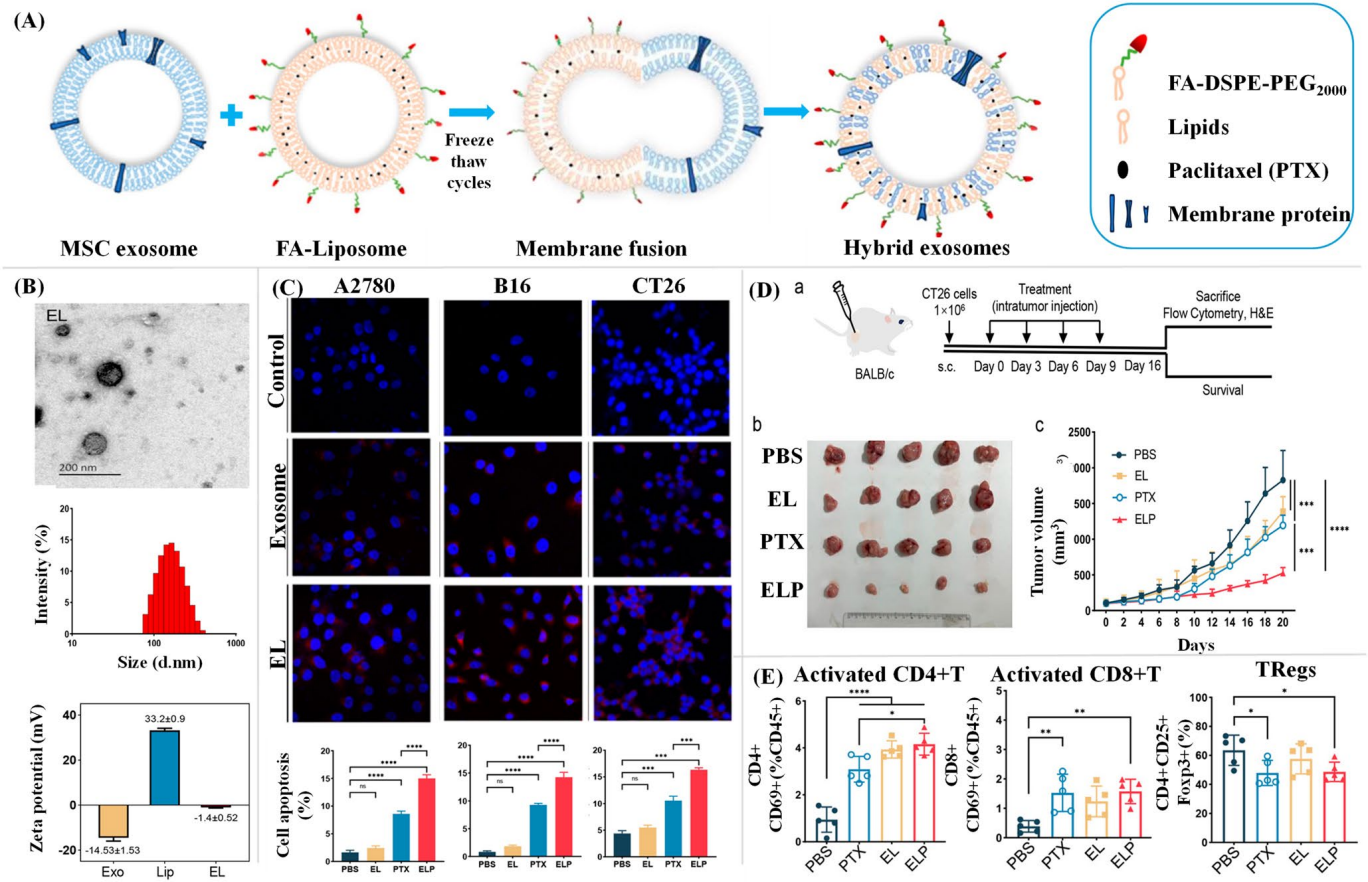
Active hybrid exosome drug delivery system for paclitaxel. (A) Scheme of PTX‐loaded hybrid exosomes (PTX‐HEs) development with paclitaxel. (B) Morphology, size, and zeta potential of PTX‐HEs. (C) In vitro cellular uptake and cell apoptosis, assays revealed the active internalization of EL and enhanced apoptotic activity of PTX‐HEs over free exosomes and free PTX. (D) In vivo antitumor effect of PTX‐HEs against CT26 tumor‐induced mouse model. PTX‐HEs showed significantly anti‐cancer activity than the free PTX. (E) Effect of PTX‐HEs on in vivo intra‐tumoral immunity activation. (Reprinted with permission from Wang et al. (2024). Copyright 2024 MDPI).

Wang et al. developed a novel approach to cancer therapy by developing an engineered exosome. The authors explore the potency of hybrid exosomes created by fusing mesenchymal stem cell (MSC) derived exosomes with folate‐modified liposomes containing paclitaxel (PTX) to enhance the drug load and achieve the specific site targeting ability. This work employed the ultracentrifuge method to isolate exosomes from mesenchymal stem cells (MSCs). The folate‐functionalized liposome has been developed, and the paclitaxel (PTX) has been loaded. The PTX‐loaded hybrid exosomes (PTX‐HEs) were formulated utilizing a freeze–thaw technique (Figure 13A). Higher paclitaxel load was observed in the PTX‐HEs when compared to the PTX‐exosomes (Figure 13B). The hybrid exosomes enhanced drug delivery and demonstrated a prolonged release profile, essential for maintaining therapeutic levels inside the tumor microenvironment (Figure 13C). The in vivo study undertaken in the CT26 colon cancer‐bearing mice model resulted in the higher therapeutic efficacy of hybrid exosomes, indicating the enhanced therapeutic profile of HEs (Figure 13D). The authors also revealed the ability of PTX‐HEs to enhance drug delivery and their ability to alter the tumor microenvironment by activating CD4+ and CD8+ T cells, polarizing macrophages towards the M1 phenotype, and reducing regulatory T cells (TRegs), thus augmenting the antitumor immune response (Figure 13E). The study presents persuasive evidence that hybrid exosomes constitute a promising platform for cancer therapy. They efficiently encapsulate and deliver chemotherapeutic agents, improve drug stability, enhance targeting specificity, and beneficially alter the tumor microenvironment. These diverse benefits establish hybrid exosomes as a key advancement in drug delivery systems, providing a novel approach that can be customized for different treatment options for cancer (Wang et al. 2024).

**FIGURE 14:**
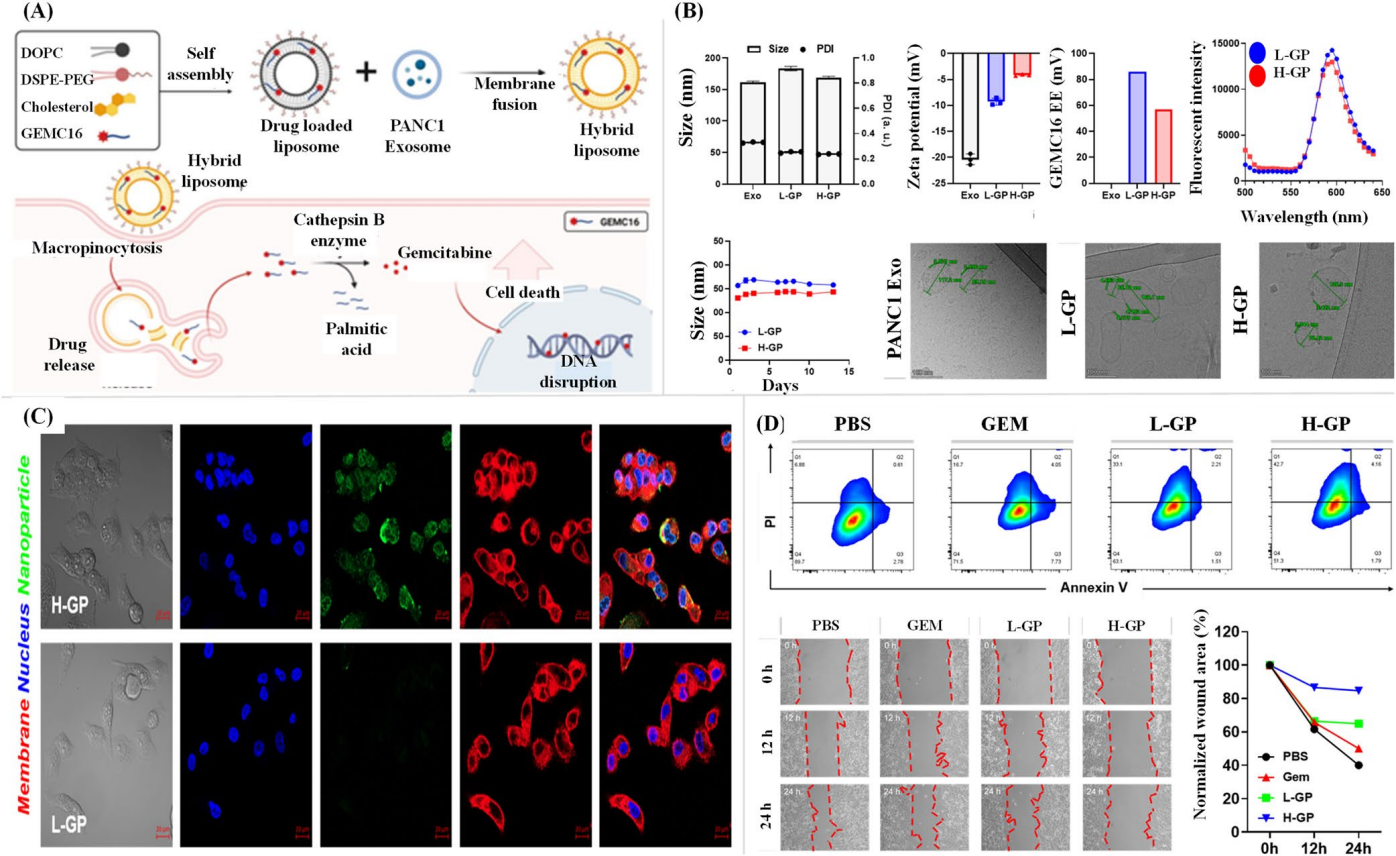
Gemcitabine prodrug‐loaded EVs for PDAC treatment. (A) Overview of HEVs preparation and its targeting mechanism in cells. (B) Size, Zeta potential, stability, and morphology assessment of NPs. (C) Cellular uptake evaluation by CLSM in PANC1 cell line. (D) In vitro antitumor activity of nanoparticles. (Reprinted with permission from Kim, Park, et al. (2024). Copyright 2024 ACS Publications).

**FIGURE 15:**
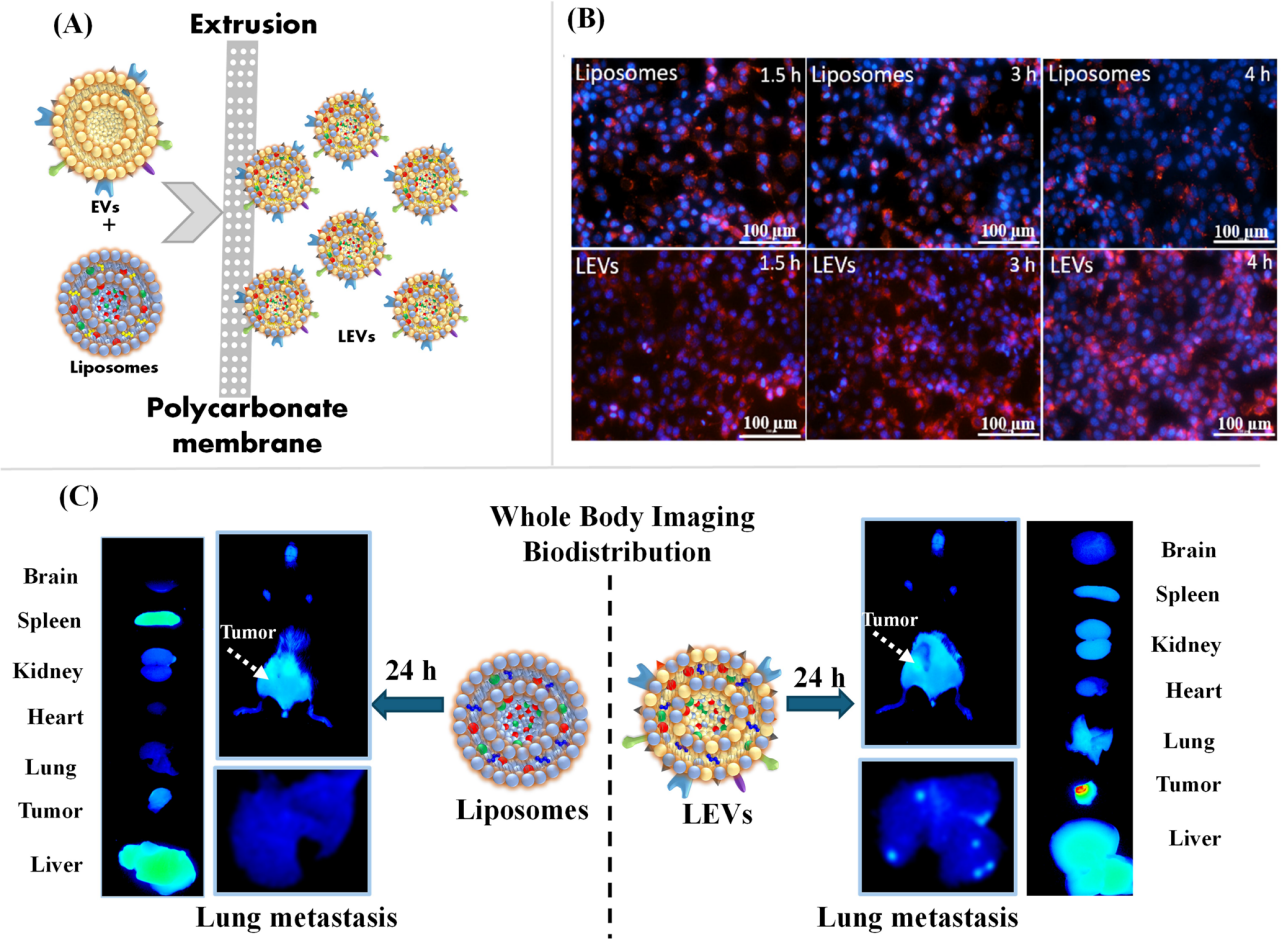
4T1 tumor‐derived EVs hybridized with liposome development to maximize the nanoparticle tumor delivery. (A) Schematic representation of hybridization. (B) In vitro cellular internalization of liposomes and Liposome hybridized EVs (LEVs) labeled with Rh‐B incubated 4T1 cells. Results showed maximum internalization of LEVs than the control liposomes into 4T1 cells. (C) In vivo NIR fluorescence imaging of 4T1 tumor‐bearing mice injected with DiR‐loaded LEVs and liposomes. (Reprinted with permission from Sulthana et al. (2024). Copyright 2024 The Royal Society of Chemistry).

The exosomes derived from tumor cells and immune‐based cells exert their functions and target the interested sites by utilizing their parental functional proteins. Kim and his research group derived the exosomes from pancreatic cancer cells and hybridized these exosomes with the liposome that is already loaded with Gemcitabine chemotherapeutic drug (Figure 14; Kim, Park, et al. 2024). This hybrid nanoplatform is intended to improve gemcitabine's treatment efficacy against pancreatic ductal adenocarcinoma (PDAC). By combining EVs isolated from PANC1 cells with gemcitabine‐palmitic acid prodrug‐loaded liposomes, the study takes advantage of EVs' innate targeting capabilities and the controlled drug release of liposomal carriers. The paper carefully describes how hybridized vesicles loaded with gemcitabine‐palmitic acid NPs targeted pancreatic cancer cells using exosomes' natural homing properties. In murine and human pancreatic cancer cell lines, hybridized vesicles loaded with gemcitabine‐palmitic acid had much higher cellular uptake than liposomal gemcitabine‐palmitic acid. Exosomes exploit the macropinocytosis pathway, which is elevated in PDAC cells with certain genetic mutations, in order to improve absorption. Compared to liposomal gemcitabine‐palmitic acid and free gemcitabine, hybridized vesicles loaded with gemcitabine‐palmitic acid had lower IC50 values and higher enhanced apoptosis rates against cancer cells. The discussion section of this research work thoughtfully explores the implications of these findings, suggesting that the hybrid NPs could overcome the traditional limitations of gemcitabine treatment, such as rapid systemic clearance and poor cellular uptake. This study suggests that hybridized vesicles loaded with gemcitabine‐palmitic acid could improve PDAC treatment outcomes by enhancing the drug's bioavailability and targeting ability. The hybrid approach exhibits improved cellular absorption, longer circulation time, and higher tumor accumulation, addressing the difficulties of drug resistance and systemic toxicity associated with traditional gemcitabine treatment. In vivo investigations in PDAC models show considerable tumor suppression and better survival rates, underlining the platform's potential for clinical use (Figure 14; Kim, Park, et al. 2024). Similarly, our research group has demonstrated an engineered hybrid system generated by re‐engineering EVs derived from mouse breast cancer (4T1 cells) with synthetic liposomes. The system was routinely compared for its cancer‐targeting potential in the mice bearing 4T1 tumors. Compared to synthetic liposomes, re‐engineered liposome hybridized EVs (LEVs) showed enhanced tumor accumulation, thereby putting it as one of the potential strategies to maximize tumor delivery (Figure 15). These studies highlight biomimetic systems evolved by combining vesicles from unique biogenesis with synthetic nanoparticles as a promising technique for precision cancer therapy and diagnosis (Sulthana et al. 2024).

## 5 Summary and Future Outlook

With the insertion of nanotechnology back in 1995 in the clinic (DOXIL, the first FDA‐approved liposomal Doxorubicin nano‐drug), a nanoparticle‐based drug delivery system has emerged as a controlled‐release technology that alters drug pharmacokinetics and takes advantage of the pathophysiology of disease to maximize drug delivery. Lessons learned from decades of research in nanotechnology, we are now moving forward with precision therapy using nanomedicine as a theranostic agent. However, it remains a challenge in these rapidly evolving cellular environments. Mutation in genes, drug resistance, deep tissue inflammation, altered targets, etc., are major limitations that demand continuous research in these fields. The uniqueness in NPs systems is their ease in tailoring them to take advantage of diseased pathologies such as vascular permeability, hypoxia, pH, and microenvironment. For example, NPs are engineered as a stimulus‐sensitive drug delivery system that enhances drug release in tumor‐acidic environments. Learning from synthetic NPs systems, the next generation of NPs evolves as biomimetic NPs, which we have discussed in this review with a focus on merging synthetic and biogenic systems as a hybrid nanoplatform technology.

EVs are nanoscale vesicles mostly found in the extracellular space of a wide range of cell types, including but not limited to mast cells, epithelial and endothelial cells, dendritic cells, astrocytes, and cancer cells. They carry functional properties from their mother cells, which depend on their biogenesis, resulting in various classes of EVs. The first class of EVs is exosomes, which are of endosomal origin; the second class is microvesicles originating from the plasma membrane; and the third class of EVs originates from apoptotic bodies. Due to their distinctive origins, EVs are well‐trained to target their destination with the help of membrane‐bound proteins to trigger a response. For example, a large amount of transferrin (Tf) on the surface of cancer cells can bind to transferrin receptors (TfR) naturally present on the surface of EVs. Similarly, cancer cell‐derived EVs localize into tumors more efficiently. While there have been a number of reports explaining the response of EVs, we are in a very preliminary stage to take EVs to the clinic. Limitations such as their isolation yield, variation in functional properties, heterogeneity in isolated populations, and, more importantly, their colloidal stability and scalability are hindering factors to take EVs to the next level. Therefore, it is highly essential to look for alternatives, which we have summarized in this review as a hybrid platform technology by re‐engineering EVs with a synthetic NP system. While encouraging results have been observed, questions such as the optimum ratio of synthetic and biogenic systems, stability of the engineered system, in vivo tracking, purification, and payload loading need to be studied to streamline the study towards biomedical applications.

## Author Contributions

**Viswanathan Sundaram:** conceptualization (supporting), formal analysis (equal), investigation (equal), methodology (equal), software (equal), validation (equal), visualization (equal), writing – original draft (equal), writing – review and editing (equal). **Santosh Aryal:** conceptualization (lead), formal analysis (lead), funding acquisition (lead), investigation (equal), project administration (equal), resources (lead), software (equal), supervision (equal), visualization (lead), writing – original draft (lead), writing – review and editing (lead).

## Conflicts of Interest

The authors declare no conflicts of interest.

## Related WIREs Articles

Isolation and characterization of extracellular vesicles and future directions in diagnosis and therapy

## Data Availability

Data sharing is not applicable to this article as no new data were created or analyzed in this study.
